# Molecular profiling identifies distinct subtypes across *TP53* mutant tumors

**DOI:** 10.1172/jci.insight.156485

**Published:** 2022-12-08

**Authors:** Xin Chen, Tianqi Liu, Jianqi Wu, Chen Zhu, Gefei Guan, Cunyi Zou, Qing Guo, Xiaolin Ren, Chen Li, Peng Cheng, Wen Cheng, Anhua Wu

**Affiliations:** 1 Department of Neurosurgery, Shengjing Hospital of China Medical University, Shenyang, Liaoning, China.; 2 Department of Neurosurgery, The First Hospital of China Medical University, Shenyang, Liaoning, China.; 3 Department of Neurosurgery, Shenyang Red Cross Hospital, Shenyang, Liaoning, China.; 4 Department of Orthodontics, Stomatological Hospital of China Medical University, Shenyang, Liaoning, China

**Keywords:** Genetics, Immunology, Immunotherapy, p53

## Abstract

Tumor protein 53 mutation (*TP53mut*) is one of the most important driver events facilitating tumorigenesis, which could induce a series of chain reactions to promote tumor malignant transformation. However, the malignancy progression patterns under *TP53* mutation remain less known. Clarifying the molecular landscapes of *TP53mu*t tumors will help us understand the process of tumor development and aid precise treatment. Here, we distilled genetic and epigenetic features altered in *TP53mut* cancers for cluster-of-clusters analysis. Using integrated classification, we derived 5 different subtypes of *TP53mut* patients. These subtypes have distinct features in genomic alteration, clinical relevance, microenvironment dysregulation, and potential therapeutics. Among the 5 subtypes, COCA3 was identified as the subtype with worst prognosis, causing an immunosuppressive microenvironment and immunotherapeutic resistance. Further drug efficacy research highlighted olaparib as the most promising therapeutic agents for COCA3 tumors. Importantly, the therapeutic efficacy of olaparib in COCA3 and immunotherapy in non-COCA3 tumors was validated via in vivo experimentation. Our study explored the important molecular events and developed a subtype classification system with distinct targeted therapy strategies for different subtypes of *TP53mut* tumors. These multiomics classification systems provide a valuable resource that significantly expands the knowledge of *TP53mut* tumors and may eventually benefit in clinical practice.

## Introduction

Tumor protein 53 (*TP53*) acts as a tumor-suppressor by inducing cell cycle arrest, cellular senescence, DNA repair, apoptosis, and changes in metabolism (1, 2). Frequent mutations in *TP53* in human cancers were proposed for the first time by Vogelstein et al. (3–5). Mutations in this gene are associated with several human cancers, including hereditary cancers such as Li-Fraumeni syndrome (6). *TP53* mutation (*TP53mut*) is universal across cancer types, and mutation rates of this gene range from < 5% to > 90% (7). *TP53mut* occurs mostly in the central DNA-binding domain (DBD), mainly in the form of missense mutations (87.9%) (7). These mutations inhibit the binding of protein-coding *TP53* to its target DNA sequences and, thus, prevent the transcriptional activation of these genes. Outside this region, however, missense mutations account for only about 40%, the majority of mutations being nonsense or frameshift mutations. Therefore, extensive mutation patterns of *TP53* in different situations lead to different molecular biological features during tumorigenesis. For example, the R249S mutation observed in hepatocellular carcinoma due to G-to-T transversions is associated with exposure to aflatoxin, and the R213* mutation in melanoma is associated with the C-to-T transition signature of ultraviolet (UV) mutagenesis (8). Studies conducted over the past 25 years suggest that certain mutant *TP53* alleles have “gain-of-function” properties such as enhanced invasion and metastasis. However, in some cases, certain mutants enhance drug resistance, epigenetic reprogramming, or angiogenesis (8, 9). In addition, *TP53mut* is considered the most widely used molecular marker for tumor classification and determination of the appropriate treatment. For now, previous efforts have mostly attempted to clarify the molecular, biological, and clinical differences between tumors with or without *TP53mut*; a huge clinical and molecular heterogeneity continues to be present in large sample size on tumors with *TP53mut* (10). Therefore, studies investigating malignancy progression and therapeutic response patterns in *TP53mut* should be urgently conducted.

The 2-hit hypothesis, first formulated by Alfred G. Knudson, states that malignant transformation of a normal cell into a cancer cell is usually caused by inactivation of tumor suppressor genes either through mutations or other epigenetic silencing (11). More than 91% of *TP53mut* cancers exhibit loss of the second allele due to mutation, chromosomal deletion, or copy-neutral loss of heterozygosity (10). The most typical configuration of *TP53mut* is a single *TP53mut* with loss of the remaining *TP53* allele due to a deletion on chromosome band 17p. Other less-common configurations include mutations in both *TP53* alleles or mutation in 1 allele and retention of the second WT allele. However, mutations in both *TP53* alleles do not always result in cancer, suggesting that additional molecular alterations are required for malignant transformation. In some cancers, *TP53* mutations often occur together with the activation of *KRAS* mutations or *MYC* amplifications (12, 13). *TP53muts* are often associated with high rates of copy number variation (CNV), as observed in ovarian carcinoma and AML with complex karyotype (14). However, the key molecular events that determine the next step in tumor development in *TP53mut* have not been systematically studied, to date. Further analysis of the following molecular change as the second hit in *TP53mut* tumor will help us to understand the process of tumor development and enable the development of new therapies.

In recent years, immune checkpoint blockade (ICB) therapy has demonstrated unprecedented rates of durable clinical benefit in patients with various cancers (15–24). Although patients with *TP53mut* often have a poor prognosis, results from a clinical trial KEYNOTE-001 showed that in patients with non–small cell lung cancer (NSCLC) treated with programmed death 1 (PD-1) inhibitors, immunotherapy worked better in patients with *TP53mut* (25). However, these beneficial effects were not observed in all patients with *TP53mut*. The associations between different mutation patterns of *TP53* and ICB therapy response have also been investigated, and this yielded controversial results. Evidence proved *TP53* missense and nonsense mutations were both associated with increased tumor mutational burden (TMB) and neoantigen levels, suggesting a better immunotherapy response. While survival analysis revealed that only patients with *TP53* missense mutations got better clinical benefit from anti–PD-1/L1 therapy (26). Therefore, in addition to mutation patterns, there exist other intratumoral factors associated with immunotherapy response in *TP53mut* patients. The criteria for selecting and treating patients with *TP53mut* resistant to ICB need to be established.

To address the above issues, we performed an integrative multiplatform analysis of patients with *TP53mut* and obtained a sum of 5 cluster-of-clusters analysis (COCA) subtypes. Each subtype exhibits distinct genetic and epigenetic features that lead to different biological functions and clinical outcomes. In particular, COCA3 tumors have an immunosuppressive microenvironment that contributes to their unfavorable prognosis and resistance to ICB therapy. In addition, drug screening, as well as in vitro and in vivo experiments, showed that olaparib is a promising agent for the treatment of COCA3 tumors. Overall, the results of our integrated analysis provide potentially novel insights into the molecular events and tumor management of patients with *TP53mut* using a multiomics classification system.

## Results

### Data processing.

To systematically characterize the features of *TP53mut* tumors, we developed a method for data processing, which was shown in Supplemental Figure 1 (supplemental material available online with this article; https://doi.org/10.1172/jci.insight.156485DS1). We reviewed the molecular and clinical information of 9,104 patients from The Cancer Genome Atlas (TCGA) project. The frequency of *TP53mut* was approximately 35.9% in all tumors. The frequency of *TP53mut* ranged from 0% (uveal melanoma) to 91.2% (uterine carcinosarcoma) (Supplemental Figure 2A). Furthermore, we included patients with cancer who had a frequency of > 10% *TP53mut* and individuals with an intact mutation profile, somatic copy number alteration profile, DNA methylation profile, and clinical information > 50 (*n* = 18). Finally, a total of 2,773 patients with *TP53mut* tumors from 18 cancer types were enrolled in our study (Supplemental Figure 2, B–E, and Supplemental Table 2).

### Single and integrated platform analysis for TP53mut tumors.

Recently, results from studies on molecular taxonomy have provided insights into the classification of cancers other than those based on histological subtypes. To systematically explore the molecular alteration patterns in *TP53mut* tumors, we first performed unsupervised clustering analysis based on individual genomic and epigenomic data separately, involving mutation, copy number alteration, and DNA methylation. Classification results from each single-platform analysis yielded sets of 6, 13, and 6 groups of samples based on mutation, somatic copy number amplification (SCNA), and methylation data, respectively (Supplemental Figures 3–5). Therefore, the genetic and epigenetic profiles of *TP53mut* cancers showed considerable variations, which elicited different molecular classifications.

**Figure 1:**
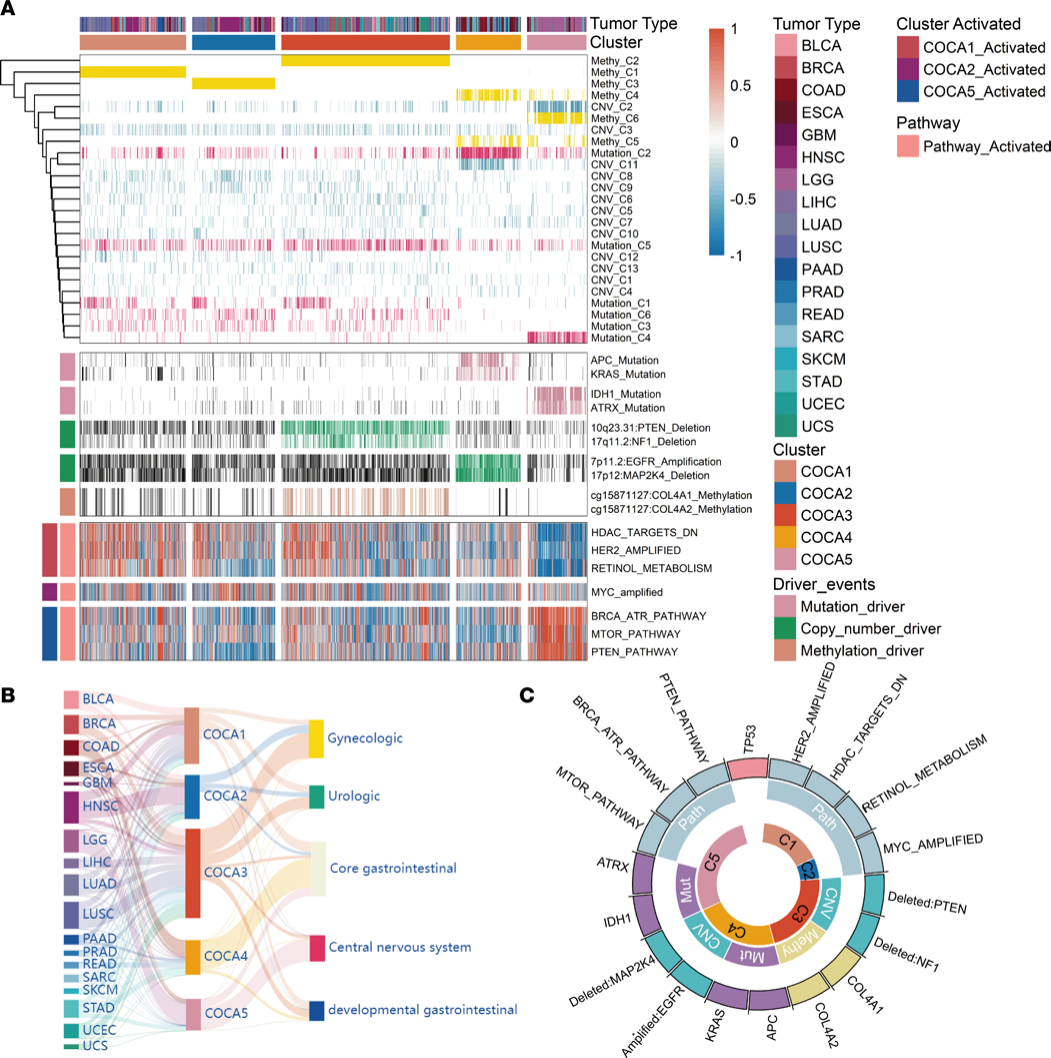
Integrated cluster-of-clusters analysis identified 5 *TP53mut* subtypes. (**A**) Integration of subtype classification from 3 omic platforms resulted in 5 major subtypes from 18 distinct cancer types. The matrix clustered by multiomics from different subtypes was depicted, and each data type is represented by a different color: mutation, yellow; copy number, blue; and DNA methylation, red. Different subtypes’ diver events are also emphasized by distinct colors. (**B**) Sankey diagram illustrating the composition of cancer types and histological origins in each cluster: left, cancer type; middle, clustering cluster; and right, histological origin. (**C**) The circle graph summarizes the driver events (mutation, copy number, DNA methylation, and activated pathway) in 5 subtypes.

To gain a more comprehensive understanding of the mechanisms underlying tumor development compared with that obtained using any single type of data, we performed an integrated subtype classification for all *TP53mut* samples based on mutation, SCNA, and methylation data. The COCA algorithm based on binary vectors from single-platform clustering results was used for analysis (Supplemental Figure 6, A and B). The integrated analyses identified 5 clusters of samples with different genomic and epigenomic features (Figure 1A, top). We found that tumor types were classified into different clusters of COCA, indicating multiomics leading to potentially novel classification independent of histopathological features in patients with *TP53mut* (Figure 1B and Supplemental Figure 6C).

### Second-step oncogenic determinant candidates of TP53mut tumor.

*TP53mut* has been well established as the first molecular alteration involved in the initiation of malignant transformation; however, the steps that follow *TP53mut* have not yet been elucidated. On the basis of the 2-hit hypothesis, we investigated the potential molecular or pathway events underlying mutations in the 5 subtypes of *TP53* in various tumor malignancies. Based on the subtypes of *TP53mu*t tumors identified by COCA, the determinants of the second step were profiled by exploring the genomic, epigenomic, and pathway features. For somatic mutations, a Pan-Cancer-12 list of 127 significantly mutated genes (SMGs) obtained using MuSiC analysis was evaluated using Fisher’s exact test in each COCA subtype. We identified the characterized gene mutations in COCA4 and COCA5 subtypes. Recurrent mutations in *APC* and *KRAS* clearly distinguished the COCA4 samples from the other groups (Figure 1A, middle). Furthermore, the distinct feature of the COCA5 subtype included mutations in *IDH1* and *ATRX*, which are considered molecular features of low-grade glioma (LGG) (Figure 1A, middle).

We then profiled the SCNA pattern of each COCA subtype by analyzing the known oncogenes or tumor suppressor genes residing in significant amplification or deletion peaks. Only genes with a positive correlation between SCNA alterations and transcriptomic expression were defined as SCNA features. We found that the deletions of *PTEN* and *NF1* were significantly enriched in the COCA3 group (Figure 1A, middle). In addition, the COCA4 subtype showed amplification of *EGFR* and deletion of *MAP2K4* (Figure 1A, middle, and Supplemental Figure 7A).

For methylation profiling, the 1,000 most viable CPG sites were selected for screening. A χ^2^ test was used to identify the CPG sites with a negative correlation with transcriptomic expression, eliciting a sum of 2 characteristic genes regulated by aberrant methylation. *COL4A1* and *COL4A2* were found to be hypermethylated in COCA3, and these 2 genes are associated with angiogenesis and immune response (27) (Figure 1A, middle, and Supplemental Figure 7B).

To further investigate the patterns of pathway activation in *TP53mut* tumors, PARADIGM inference was used to assess pathway enrichment in each COCA subtype (Figure 1A, bottom). The activating pathway of each COCA subtype was identified by pairwise comparison. The pathways of HDAC_TARGETS_DN, HER2_AMPLIFIED, and RETINOL_METABOLISM were significantly activated in COCA1. COCA2 was strongly associated with the MYC_amplified pathway. In addition, the activation of MTOR_PATHWAY, PTEN_PATHWAY, and BRCA_ATR_PATHWAY was observed in the COCA5 subtype (Figure 1A, bottom). Altogether, these results identified the genomic, epigenomic, and pathway alteration profiles based on the COCA subtype system, which implied second-step hit pattern after *TP53mut* during tumorigenesis (Figure 1C).

### Biological function and clinical importance of the COCA subtypes.

**Figure 2:**
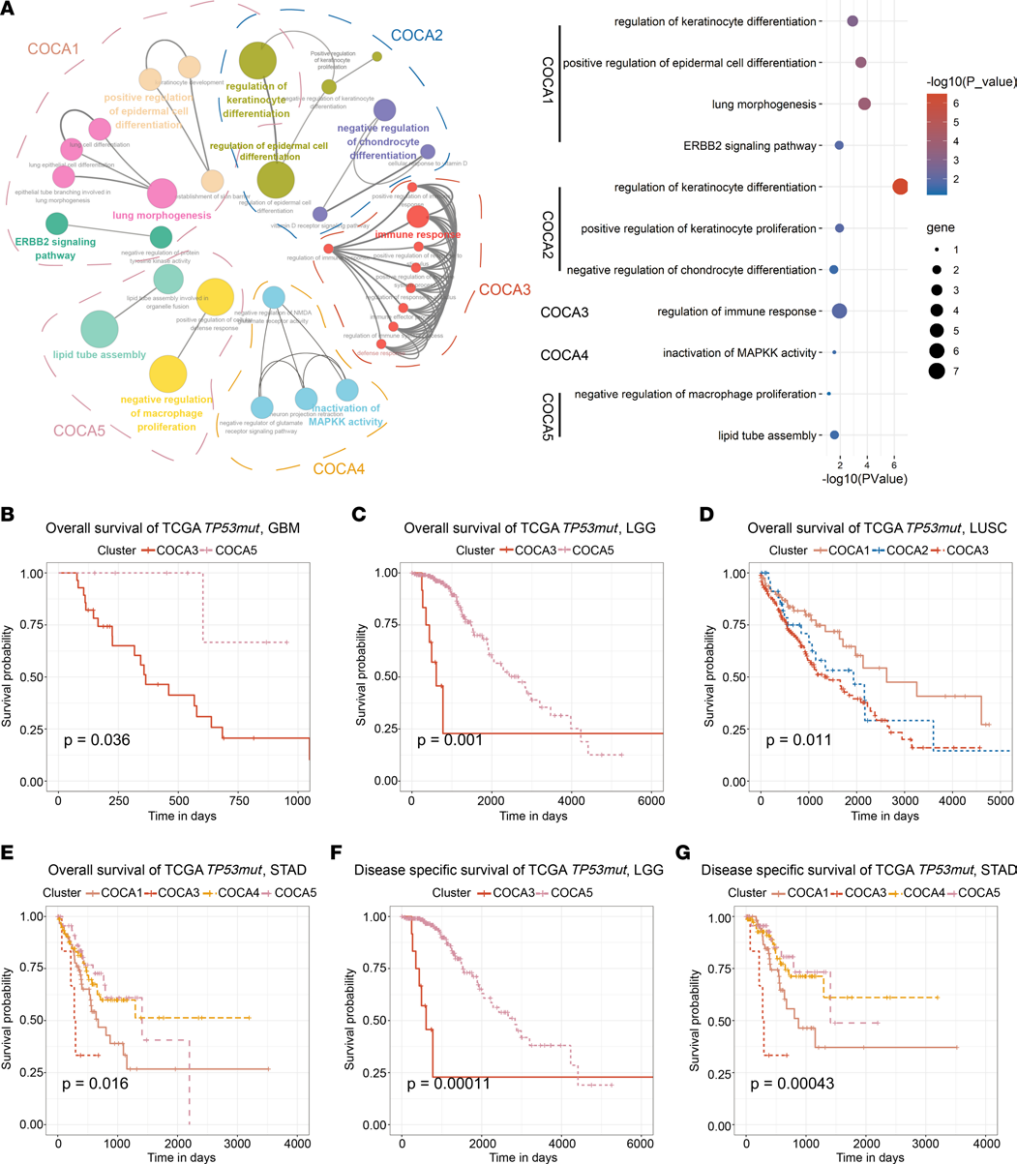
Biological functions and clinical features of COCA subtypes. (**A**) Functional annotation based on the hub genes in 5 subtypes. Left, GO analysis visualized by ClueGO. At the right, the size of the circles represents the number of genes enriched, and the color depth represented the degree of enrichment (hypergeometric test). (**B**–**E**) COCA3 subtype always exhibited an unfavorable overall survival in GBM, LGG, LUSC, and STAD (log-rank test). (**F** and **G**) COCA3 patients occupied a significantly reduced disease-specific survival in LGG and STAD (log-rank test).

We analyzed the differences in the biological phenotype of the COCA subtypes. Firstly, we identified the hub genes of each cluster based on weighted gene coexpression network analysis (WGCNA). Secondly, gene ontology (GO) analyses were performed based on the hub genes using Cytoscape, which identified the predominant biological phenotypes for COCA subtypes (Figure 2A). COCA1 was characterized by the enrichment of the ERBB2 signaling pathway, regulation of keratinocyte differentiation, and lung morphogenesis, which were highly consistent with the HER2_AMPLIFIED pathway activation in COCA1 (Figure 1A, bottom). COCA2 was enriched by the negative regulation of differentiation and positive regulation of proliferation, which were associated with the MYC_amplified pathway activation (Figure 1A, bottom). COCA3 was highly relevant to immune response, consistent with the important role of deletions in *PTEN* and *NF1* in facilitating tumor immune evasion (Figure 1A, middle). COCA4 was recognized by the inactivation of mitogen-activated protein kinase (MAPK) activity, consistent with the deletion in *MAP2K4* (Figure 1A, middle). COCA5 was associated with lipid metabolism and negative regulation of macrophage proliferation, and this association was in accordance with the MTOR and PTEN pathway activation (Figure 1A, bottom). Therefore, we identified enriched biological phenotypes for COCA subtypes, consistent with their molecular and pathway alterations.

Furthermore, we assessed the clinical relevance of the 5 clusters. First, we performed survival analysis in *TP53mut* tumors stratified by 5 COCA clusters and found the prognostic significance provided by subtyping in overall survival (OS) and disease-specific survival (DSS) (Supplemental Figure 9, A and B). Subsequently, we explored the prognostic role of COCA classification within each cancer type. The COCA subtypes showed significant associations with OS and DSS for 4 and 2 cancer types, respectively (Figure 2, B–G). Meanwhile, the Cox regression analysis also suggested their close relationships with the tumor prognosis (Supplemental Figure 9, C and D). Interestingly, in these cancers, we found the COCA subtyping conferred consistent prognostic implications that COCA3 was always associated with a worse prognosis. In addition, multivariant Cox analysis in LGG tumor indicated that the COCA3 subtype was an independent prognostic indicator when adjusted for histological grade (Supplemental Table 3). In lung squamous cell carcinoma (LUSC) and stomach adenocarcinoma (STAD) tumors, the COCA3 subtype performed an independent prognostic factor when adjusted for pathologic tumor stage (Supplemental Table 3). Altogether, these results underscored the potential clinical utility of the COCA subtyping system, and COCA3 proved to be a robust subtype with poorer prognosis.

### COCA3 conferred an immunosuppressive phenotype.

**Figure 3:**
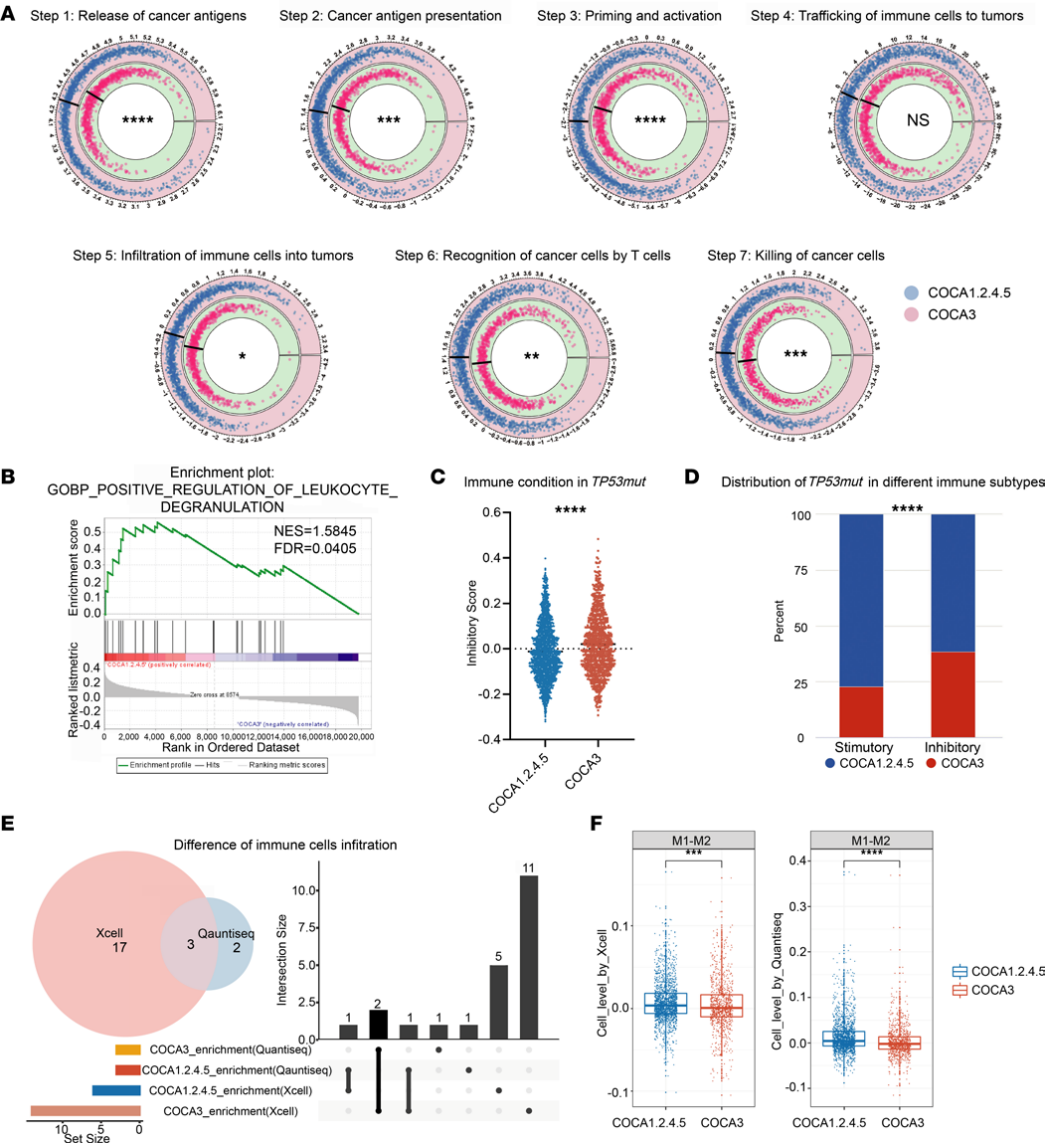
COCA3 subtype conferred an immunosuppressive microenvironment. (**A**) The 7 steps of TIP to identify dynamic changes in tumor killing between COCA3 and other 4 COCA subtypes (Student’s *t* test for steps 5 and 7, and Wilcoxon rank-sum test for other steps). (**B**) GSEA revealed that COCA3 negatively correlated with leukocyte degranulation (permutation test). (**C**) The inhibitory score estimated by ssGSEA was elevated in COCA3 subtype (Wilcoxon rank-sum test). (**D**) The identified inhibitory patients highly enriched in COCA3 subtype (χ^2^ test). (**E**) Three immune cells significantly enriched in a COCA3 subtype from quanTIseq and xCell algorithms intersected. (**F**) The contents of M1 macrophage minus M2 were decreased in COCA3 subtype (Wilcoxon rank-sum test). *P < 0.05, **P < 0.01, ***P < 0.001, ****P < 0.0001.

Considering that COCA3 conferred worse prognosis than other subtypes, we investigated the mechanisms underlying this observation. We investigated impaired immune response as a biological feature of COCA3. We evaluated the status of antitumor immunity between COCA3 and the other 4 COCAs based on the concept of a 7-step cancer immunity cycle. We found that COCA3 patients had advantages in activating antitumor immunity (first 3 steps: release of cancer antigens, cancer antigen presentation, and priming and activation). COCA3 showed disadvantages over other COCA subtypes from the aspects of immune cell infiltrating (Step 5). Therefore, immunological recognition and killing of cancer cells were significantly decreased in COCA3 tumors (Step 6 and 7) (Figure 3A).

To confirm whether COCA3 elicited a decreased antitumor response, gene set enrichment analysis (GSEA) was performed, and the results showed that COCA3 was negatively correlated with leukocyte degranulation, indicating a decreased antitumor immune response (Figure 3B). Furthermore, we summarized a total of 75 immune checkpoint genes and assessed the overall stimulatory and inhibitory status by single-sample GSEA (ssGSEA) according to immune checkpoint annotation. We found that the inhibitory checkpoint score was significantly upregulated in COCA3 (Figure 3C). After comparing the stimulatory and inhibitory scores of each patient, we divided the patients into stimulatory or inhibitory categories and observed that COCA3 was significantly enriched in the inhibitory category (Figure 3D). All these results indicate that the significant features of COCA3 included attenuated antitumor immunity, which may be caused by aberrant immune cell trafficking and infiltrating.

To analyze the content of immune cells, we identified the canonical immune cell leading to the inhibition of the ability of killing of T cells in COCA3. The significant infiltration status of immune cells calculated by the quanTIseq and xCell algorithms was assessed between COCA3 and other COCAs (Supplemental Figure 10, A and B). The results reveal that the number of B cells and CD8^+^ T cells was simultaneously increased, whereas the content of M1 macrophages minus M2 macrophages was decreased in COCA3 (Figure 3, E and F, and Supplemental Figure 10C). Further evaluation of cellular composition showed that the number of macrophages was higher than that of the other 2 cell types, indicating their crucial role during immune response (Supplemental Figure 10D). Thus, we concluded that the COCA3 subtype conferred dynamic polarization of M1 to M2 macrophages, partially leading to a poor antitumor ability in spite of CD8^+^ T cell and B cell infiltration. Altogether, the results indicate that the immunosuppressive microenvironment resulting from the polarization of M1 to M2 macrophages caused decreased antitumor immunity in patients with the COCA3 subtype.

### A classification method based on COCA features to predict immunotherapy response.

**Figure 4:**
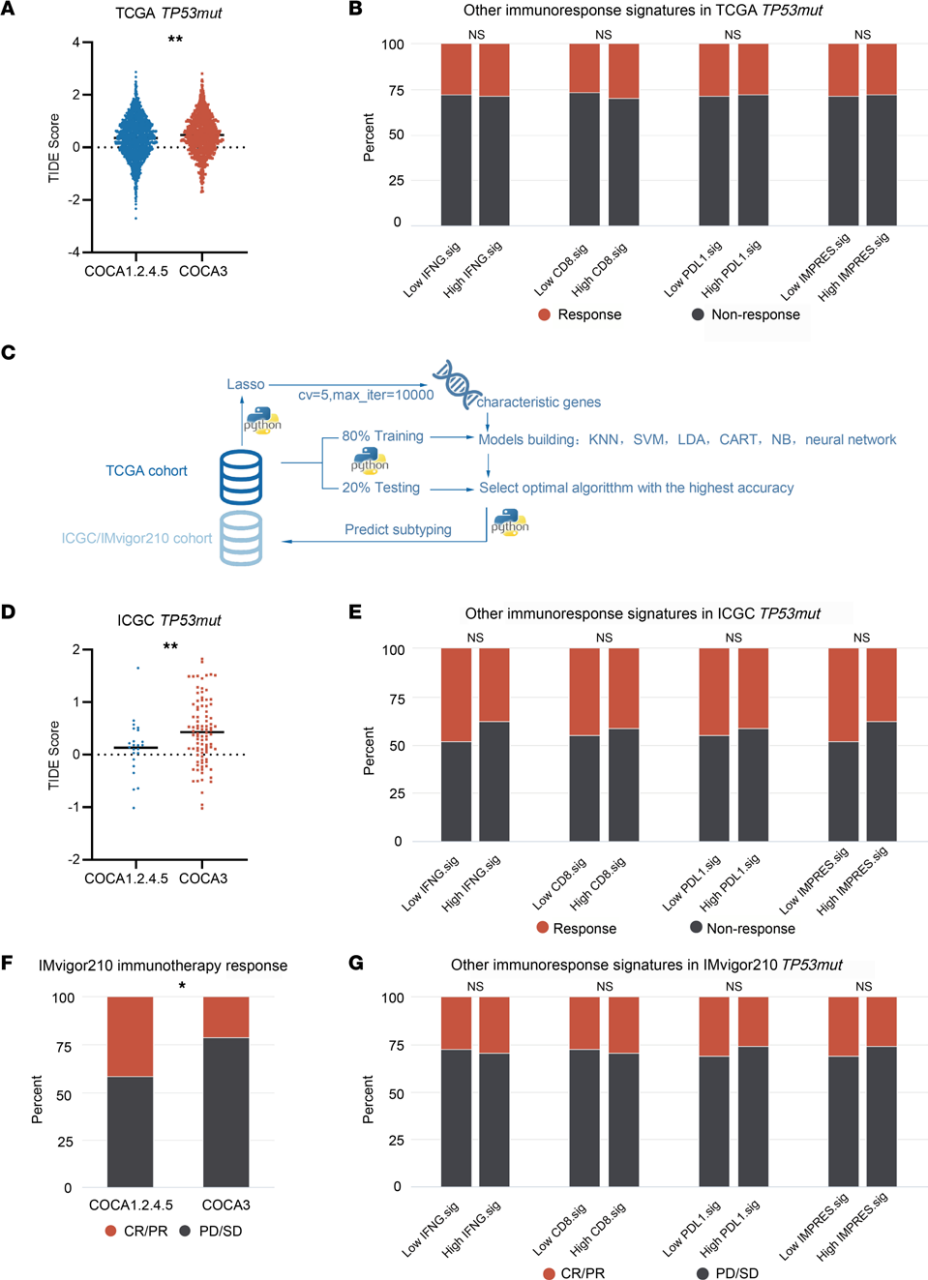
COCA subtyping predicted immunotherapeutic response. (**A**) COCA3 subtype exhibited less of a response to immunotherapy predicted by TIDE in TCGA cohort (Wilcoxon rank-sum test). (**B**) The distribution of response and nonresponse *TP53mut* patients showed no significant difference between high and low immunoresponse signatures in TCGA cohort (χ^2^ test). (**C**) The flowchart showed the process to predict COCA subtypes in ICGA and IMvigor210 cohorts. (**D**) COCA3 possessed a higher TIDE score than COCA1.2.4.5 subtype in ICGC cohort (Student’s *t* test). (**E**) The 4 immunotherapy-associated signatures could not separate the responders from nonresponders of *TP53mut* in ICGC cohort (χ^2^ test). (**F**) The immunotherapy response *TP53mut* patients (complete response/partial response [CR/PR]) was distributed more in the COCA1.2.4.5 subtype, while nonresponse ones (progressive disease/stable disease [PD/SD]) enriched in COCA3 subtype in IMvigor210 cohort (χ^2^ test). (**G**) The 4 immunotherapy signatures did not have a predictive value for immunotherapy in *TP53mut* patients in IMvigor210 cohort (χ^2^ test). *P < 0.05, **P < 0.01.

Recently, inhibition of immunological checkpoints using monoclonal antibodies that block T cell inhibitory molecules has emerged as an anticancer treatment with unprecedented and synergistic survival benefits. The ICB therapy showed remarkable efficacy in patients with *TP53mut*. Since attenuated T cell immunity was observed in COCA3, we investigated its implications in immunotherapy. We first employed the tumor immune dysfunction and exclusion (TIDE) algorithm to evaluate clinical response to immunotherapy. We compared the response effect in COCA3 with that in other 4 clusters. We found that COCA3 was less likely to respond to ICB compared with the other 4 clusters (Figure 4A). We used other 4 immunoresponse signatures previously reported as references in the TCGA cohort, whose high levels reflected better response to immunotherapy (Supplemental Table 5). On the basis of the median value of each signature obtained from the ssGSEA results, we separated patients with *TP53mut* into low and high groups. Unfortunately, these ICB response signatures did not work well in *TP53mut* tumors (Figure 4B). Therefore, the COCA subtyping of *TP53mut* tumors has critical clinical significance in the context of tumor immunological status and immunotherapy response.

Because of the poor prognosis and immune response of COCA3, it was of great value to accurately distinguish COCA3 tumors from other subtypes. RNA-Seq data are widely used in clinical and scientific research; therefore, we developed a method to distinguish COCA3 tumors from tumors of other subtypes based on RNA-Seq data. Machine learning methods were developed for this purpose (Figure 4C). We first performed lasso regression analysis with the optimal α value (α = 0.0015) in the TCGA mRNA-Seq cohort and selected a sum of 489 characteristic genes that could accurately separate COCA1.2.4.5 and COCA3. Subsequently, patients were divided into a training set and a validation set (80% and 20%, respectively). The training set was used to train prediction models based on these characteristic genes with several machine learning algorithms including k-nearest neighbor (KNN), support vector machine (SVM), linear discriminant analysis (LDA), classification and regression tree (CART), naive Bayes (NB), and neural network. Finally, the neural network had the highest predictive accuracy (100% in the training cohort and 95.1% in the validation cohort) and was selected to distinguish COCA3 tumors from tumors in other COCA groups.

In order to verify the clinical implications of our neural network algorithm, we used the ICGC cohort to perform the COCA classification. Firstly, we screened *TP53mut* tumors in the ICGC cohort aside from the TCGA cohort. Secondly, we recognized COCA3 tumors from the ICGC cohort using the neural network algorithm. As expected, COCA3 patients showed significantly shortened survival time (Supplemental Figure 11A). Biological analysis showed that patients with COCA3 had decreased antitumor immunity and abundant M2 macrophage enrichment (Supplemental Figure 11B). Furthermore, we calculated the TIDE score of patients with *TP53mut* in the ICGC cohort. The TIDE score was significantly increased in COCA3 tumors (Figure 4D). However, the other 4 well-established immune-response signatures did not work well in the ICGC cohort (Figure 4E).

Subsequently, our neural network algorithm was used to analyze the IMvigor210 cohort, which was a cohort from a phase II trial of atezolizumab in advanced or metastatic urothelial carcinoma (Supplemental Figure 11C). We found that COCA3 subtype was associated with a tendency of worse prognosis with a marginal statistical significance (*P* = 0.09) and an immunosuppressive microenvironment (Supplemental Figure 11, D–F). Comparison of immunotherapy response between patients with COCA3 tumors and tumors of other subtypes showed that patients with the COCA3 subtype showed a poorer effectiveness of treatment (Figure 4F). Furthermore, our results showed that COCA subtyping was the best model for predicting the response of patients with *TP53mut* to immunotherapy compared with other immune-response signatures (Figure 4G). These results indicate that the neural network algorithm could accurately recognize COCA3 tumors with ICB therapeutic resistance.

### Identification of olaparib as the most promising therapeutic agents for COCA3 tumors.

**Figure 5:**
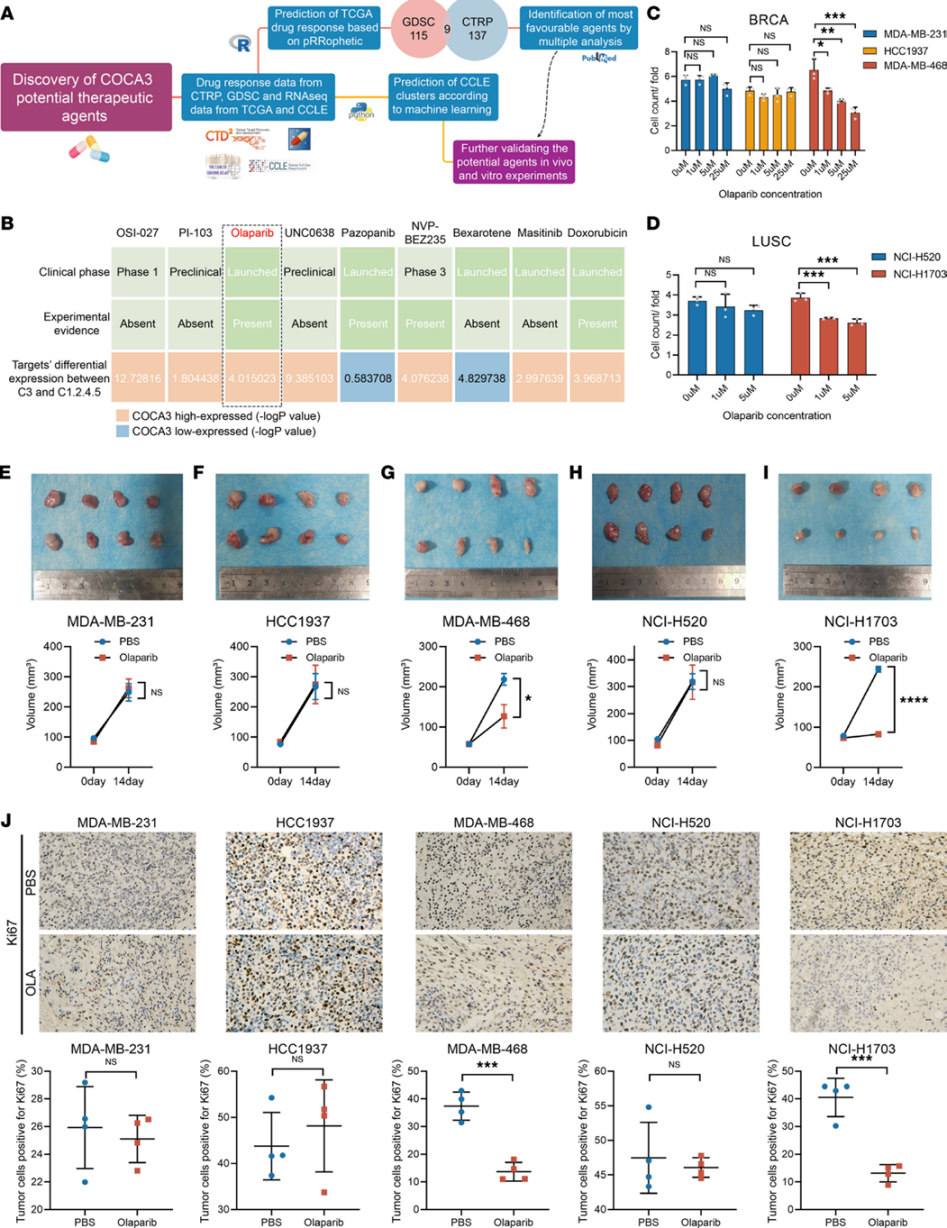
Identification and validation of olaparib as the promising agents for COCA3 subtype. (**A**) Schematic revealed the process to identify COCA3-specific agents. (**B**) Identification of olaparib and as the most promising agent for COCA3 according to the evidence from multiple sources (Student’s *t* test for OSI-027, olaparib, and pazopanib; Wilcoxon rank-sum test for other agents). (**C**) MDA-MB-468 was more sensitive to olaparib than MDA-MB-231 and HCC1937 in vitro (*n* = 3) (1-way ANOVA followed by Tukey’s multiple-comparison test). (**D**) Olaparib exhibited stronger killing ability in NCI-H1703 than that in NCI-H520 in vitro (*n* = 3) (1-way ANOVA followed by Tukey’s multiple-comparison test). (**E**–**I**) The tumor volume changes of 4 kinds of cells in tumor-bearing mice (*n* = 4) (Student’s *t* test). (**J**) The Ki67 levels stained by IHC in different tumor tissues (*n* = 4) (Student’s *t* test). Scale bars: 10 μm. *P < 0.05, **P < 0.01, ***P < 0.001, ****P < 0.0001.

Considering the poor prognosis and resistance to ICB therapy of COCA3, we attempted to screen agents with therapeutic effects against COCA3 (Figure 5A). To analyze drug efficacy in *TP53mut* tumors, we reviewed the *TP53mut* status, gene expression, and drug sensitivity profiles of cancer cell lines (CCLs) from the GDSC and CTRPv2 data sets. We selected *TP53* mutant cell lines belonging to 18 cancer types. Subsequently, drugs with unavailable data in more than 20% of the CCLs were removed. After the filtering procedure, 241 CCLs with information regarding 224 drugs were left in the GDSC data set and 209 CCLs with information regarding 441 drugs were left in the CTRP data set. Subsequently, we used the pRRophetic algorithm to estimate the AUC value of each compound for clinical samples based on the CTRP and GDSC data. Patients of drug AUC values derived from CTRP and GDSC data were then compared between COCA3 and other subtypes. We finally obtained a sum of 9 drugs with significantly lower AUC values in COCA3 than in non-COCA3 subtypes in the CTRP and GDSC data at the same time, and they was considered as COCA3-specific effective drug candidates (OSI-027, PI-103, olaparib, UNC0638, pazopanib, NVP-BEZ235, bexarotene, masitinib, and doxorubicin) (*P* < 0.05). Considering clinical implications, we conducted further multiple perspective analyses. First, we performed a comprehensive literature search in PubMed to identify the experimental and clinical evidence of these compounds in treating patients with *TP53mut* (28–31). Then, we compared the average expression levels of target-drug candidates between COCA3 and other subtypes (Figure 5B). Furthermore, we summarized the gene sets of targeted signal pathway of these 9 drugs and calculated each patient’s pathway score in COCA3 subtype by ssGSEA algorithm to represent the activity of pathway (Supplemental Table 6 and Supplemental Figure 12A). By comparing the targeted pathway scores of these drugs, we found that the activities of DNA damage repair (olaparib targeted) and DNA replication (doxorubicin targeted) were significantly higher than those of other pathways in COCA3 subtype (Supplemental Figure 12B). These activated signals in COCA3 suggested that the application of olaparib or doxorubicin might create potential therapeutic effects for the COCA3 subtype. In summary, olaparib together with doxorubicin were identified as the promising agents for treatment of patients with the COCA3 subtype.

Furthermore, we used the neural network algorithm to classify CCLs based on RNA expression data. We found that 85 and 157 CCLs were grouped into COCA3 and COCA1.2.4.5*,* respectively (Supplemental Table 7). Three BRCA-CCLs (MDA-MB-468 [COCA3], HCC1937 [COCA1.2.4.5], and MDA-MB-231 [COCA1.2.4.5]) and 2 LUSC-CCLs (NCI-H1703 [COCA3] and NCI-H520 [COCA1.2.4.5]) were selected for further validation.

Firstly, we compared the effects of different COCA subtypes of CCLs on macrophage polarization. Consistently, after cocultured with COCA3 cell lines (MDA-MB-468 and NCI-H1703), we found that the M0 cells were more polarized toward M2 cells than those cocultured with non-COCA3 cell lines (MDA-MB-231, HCC1937, and NCI-H520) by flow cytometry (Supplemental Figure 13A) and reverse transcription PCR (RT-PCR) (Supplemental Figure 13B). These experiments all indicate the significant role of COCA3 cells in forming an immunosuppresive microenvironment.

We then examined whether olaparib and doxorubicin were specifically effective against COCA3 tumors. Our results reveal that olaparib and doxorubicin significantly inhibited the cellular proliferation in MDA-MB-468 and NCI-H1703 cells but did not markedly affect the proliferation of MDA-MB-231, HCC1937, and NCI-H520 cells (Figure 5, C and D, and Supplemental Figure 13C). Considering the wide clinical applications of olaparib in different types of tumors than doxorubicin (32–35), we chose olaparib for further in vivo experiments. The results of in vivo studies show that olaparib had remarkable therapeutic efficacy in MDA-MB-468 and NCI-H1703 cell models rather than MDA-MB-231, HCC1937, and NCI-H520 cell models (Figure 5, E–I, and Supplemental Figure 13D). Olaparib treatment led to attenuated Ki67 expression staining in MDA-MB-468 and NCI-H1703 tissues (Figure 5J). Altogether, we identified olaparib as the most promising drug specific for COCA3 tumors, suggesting the significance of our classification methods in further precision treatment of *TP53mut* tumors.

### Medication options for different subtypes of TP53mut tumors through in vivo experiments validation.

**Figure 6:**
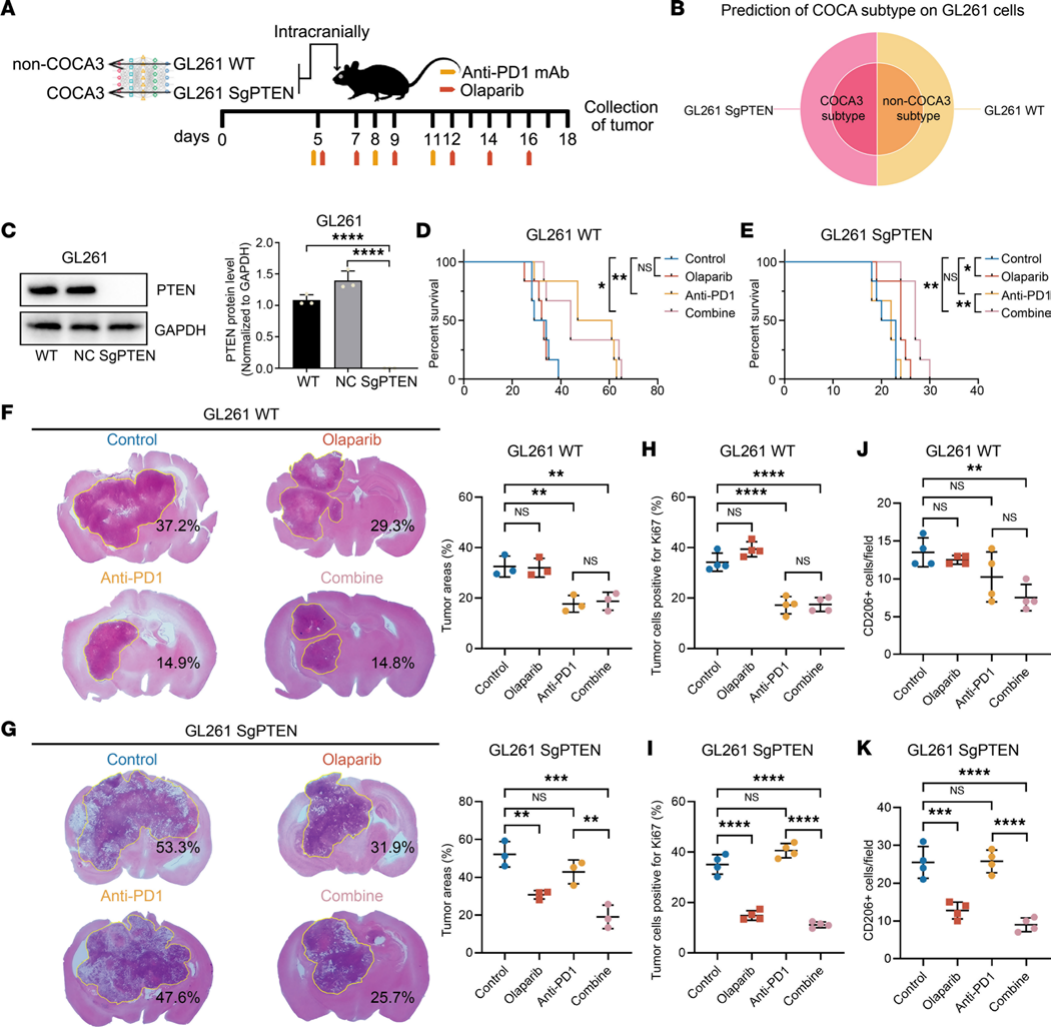
Treatment implications in different subtypes of *TP53mut* tumors. (**A**) The schematic illustration of prediction of GL261 cells and different treatments in GL261-bearing mice. (**B**) The prediction results of GL261 cells through neural networks. (**C**) Western blot revealed the KO efficiency of PTEN gene in GL261 cells (*n* = 3) (1-way ANOVA followed by Tukey’s multiple-comparison test). (**D** and **E**) Survival of GL261 WT-bearing and GL261 SgPTEN-bearing mice under different treatments (*n* = 6) (log-rank test). (**F** and **G**) H&E staining from GL261 WT-bearing and GL261 SgPTEN-bearing mice after various treatments (*n* = 4) (1-way ANOVA followed by Tukey’s multiple-comparison test). (**H**–**K**) IHC staining of Ki67 and CD206 from GL261 WT-bearing and GL261 SgPTEN-bearing mice after various treatments (*n* = 4) (1-way ANOVA followed by Tukey’s multiple-comparison test). *P < 0.05, **P < 0.01, ***P < 0.001, ****P < 0.0001.

To explore the crucial value of our classification system for the precise treatment of *TP53mut* tumors, we selected murine cell lines for further studies in vivo. Through systematic investigation of murine cell lines, combined with RNA-Seq results, we selected GL261 murine cell as a representative *TP53mut* tumor cell line predicted as non-COCA3 subtype (Figure 6, A and B) (36). In order to establish a COCA3 cell line model, considering our previous findings that *PTEN* deletion was one of the key molecule events responsible for the classification of the COCA3 subtype, we tried to knock out PTEN to see whether GL261 would convert to COCA3 subtype. We then knocked out PTEN on GL261 cells using CRISPR technology and verified the KO efficiency by Western blot (Figure 6C; see complete unedited blots in supplemental material). As expected, GL261 SgPTEN cell was predicted as COCA3 subtype, suggesting the driving event (PTEN deletion) we unearthed was indeed the key factor shaping the formation of the COCA3 subtype (Figure 6B). Hence, we successfully established a paired COCA3 (GL261 WT) and non-COCA3 (GL261 SgPTEN) cell models for further investigation.

To verify the drug benefits of different subtypes of cells, we performed GL261 WT- and GL261 SgPTEN-bearing C57BL/6 mice treated with anti–PD-1 antibody alone, olaparib alone, or their combination (Figure 6A). We found the treatment of olaparib could prolong the survival of GL261 SgPTEN-bearing mice and reduce the tumor size (Figure 6, E and G), while this benefit did not apply to GL261 WT-bearing mice (Figure 6, D and F). On the other hand, after treatment of anti–PD-1 antibody alone, GL261 WT-bearing mice survived longer with smaller tumor size (Figure 6, D and F). Even though anti–PD-1 antibody alone could not improve the GL261 SgPTEN-bearing mice outcome, after combination of the olaparib and anti–PD-1, immunotherapy-resistant mice became treatment sensitive and had significantly longer survival (Figure 6, E and G). The Ki67 IHC staining–reflected tumor proliferative capacity also confirmed these conclusions (Figure 6, H and I, and Supplemental Figure 14, A and B). Further IHC staining of CD206 (marker of M2 macrophages) revealed the increased M2 macrophages infiltration in GL261 SgPTEN-bearing mice than in GL261 WT-bearing ones (Control groups) (Figure 6, J and K, and Supplemental Figure 14, A and B). For GL261 SgPTEN-bearing mice, the treatment of olaparib restrained the infiltration of M2 macrophages, and the combination treatment also significantly decreased the M2 macrophages compared with only anti–PD-1 treatment (Figure 6K); this was also a reason olaparib could increase the sensitivity of anti–PD-1 therapy. Altogether, we established effective treatment strategies for different subtypes of *TP53mut* tumors based on our classification system.

## Discussion

**Figure 7:**
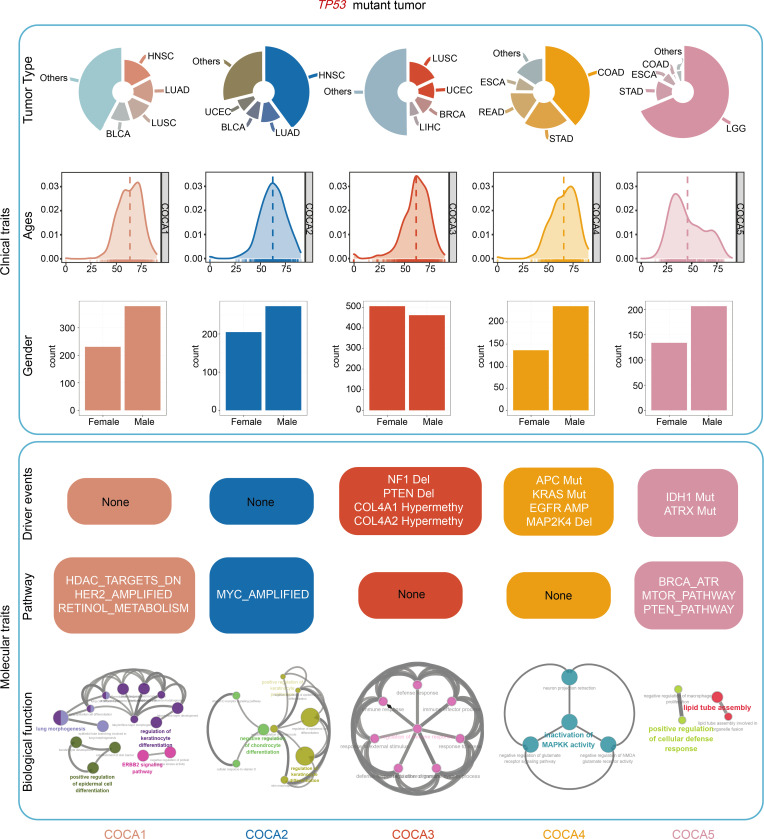
Overview of 5 major subtypes of *TP53mut* patients. A summary of 5 *TP53mut* subtypes’ clinical features including tumor ratio, age and sex together with molecular traits including driver events, activated pathway and biological function.

Several studies suggest that *TP53mut* has widespread effects that facilitate malignant transformation (1, 2). To date, most studies have focused on determining the distinguishing characteristics between tumors with and without *TP53mut* (6). Despite a large number of *TP53mut* tumors, substantial variations exist in individual tumors. Therefore, characterization of the molecular and clinical patterns in *TP53mut* tumors is of great importance to provide insights for the management and treatment of patients with *TP53mut*. In this study, we performed an integrated multiplatform analysis of pan-*TP53mut* patients in 18 cancer types. We developed a 5-subtype classification system with distinct clinical and biological characteristics, which provided potentially novel insights for the management of tumors with *TP53mut* (Figure 7).

The 2-hit theory of carcinogenesis showed the occurrence and development of tumors from the perspective of gene mutations (11). *TP53mut* has been shown to be one of the earliest molecular hits during tumorigenesis, facilitating the occurrence of other oncogenic alterations (37). A number of characteristics unique to *TP53mut* tumors have been identified, suggesting that tumors with or without *TP53mut* are different entities from both biological and clinical perspectives. However, to date, limited information is available on the oncogenic alteration patterns after *TP53mut* and their impact on biological phenotype and clinical outcome. Here, we developed a comprehensive classification system based on mutation, SCNA, and methylation profiles (Figure 7). The results of further analyses revealed specific biological functions in the 5 subtypes. These significant features may be attributed to molecular alterations caused by *TP53mut*. In particular, COCA1 was characterized by HER2_AMPLIFIED leading to an epithelial differentiation function (Figure 7). *MYC* amplification was significantly activated in COCA2, which was associated with tumor cell proliferation (Figure 7). COCA3 was characteristically formed by the deletion of *NF1* and *PTEN* with hypermethylation of *COL4A1* and *COL4A2*, and COCA4 formation was driven by mutations in *APC* and *KRAS*, consistent with the previous reports of regulation of TP53 in its downstream molecules such as APC and KRAS. The mutations of *IDH1* and *ATRX* caused the formation of COCA5. Moreover, COCA5 activated the PTEN and MTOR pathways, resulting in lipid metabolism and cellular defence response. Therefore, these key molecules played a role in the development of *TP53mut* and its various subtypes. Altogether, the COCA classification system may be of fundamental importance in clarifying the oncogenic alteration patterns and relevant biological and clinical implications in *TP53mut* tumors.

Despite decades of research, effective treatments for *TP53mut* cancers have not been established, thus far (38). One of the most vital reasons is that the heterogeneity of *TP53mut* tumors results from different combinations of multiple genetic alterations (hits). The results of our integrated classification system showed that the COCA3 subtype was associated with a worse outcome in several tumor types. We found that COCA3 was associated with an immunosuppressive microenvironment particularly characterized by M2 macrophage enrichment, which may be due to specific molecular patterns (deletion of *NF1* and *PTEN*). Despite CD8^+^ T cell infiltration, high levels of M2 macrophages accompanied by inhibitory immune checkpoint enrichment impaired the ability of cancer cell destruction in COCA3 tumors.

Recent studies have identified *TP53mut* as a favorable marker for ICB therapy (39). Furthermore, scientists also found that, for *TP53mut* LUAD patients, *KRAS* mutation indicated a better clinical benefit from PD-1 inhibitors (25). This suggests that the differences in immunotherapy response also existed within *TP53mut* tumors, which should be systematically studied. In our study, we detected the COCA3 subtype of *TP53mut* patients as the least sensitive cluster for ICB through the in vivo experiments together with several databases (TCGA, ICGC, and IMvigor210 databases). More importantly, we developed an RNA-based COCA3 classifier, which provides a potentially novel guide for predicting immunotherapy for *TP53mut* tumors in the future.

Tumor-targeted therapy is an important approach to improve the effect of tumor treatments (40). Precise tumor classification is the basis for targeted therapy (41, 42). Based on this theory, we performed multiperspective drug screening and eventually selected olaparib as the most promising agent for COCA3 tumors with a poor prognosis. Olaparib is a selective PARP1/2 inhibitor that typically targets *BRCA1* or *BRCA2* mutations (43). Olaparib has been approved by the FDA for the treatment of ovarian, breast, pancreatic, and prostate cancers and has been increasingly investigated in clinical trials. For now, the therapeutic effect of olaparib in patients with *TP53mut* has not been fully established. A case report revealed that inactivation of *TP53mut* can lead to olaparib resistance, despite the presence of *BRCA* mutations (44). However, the therapeutic effect of olaparib on different subtypes of patients with *TP53mut* has not yet been established. Our study found that the COCA3 subtype responded better to olaparib. In addition, olaparib could also enhance the efficacy of anti–PD-1 therapy, which was ineffective when used alone. Therefore, this study provided guidance in determining the treatment of patients with *TP53mut* with COCA3 subtype.

Our study has some limitations. Our transcriptome-based classifier is only suitable for binary classification; the accurate identification of the 5 subtypes needs to be improved. In addition, due to lack of mutation data, the clinical validation for immunotherapy was limited. Therefore, more ICB cohorts should be investigated to confirm our results.

In conclusion, the results of our integrated study demonstrate that a multiomics classification system provided insights into the molecular alteration and tumor development patterns in *TP53mut* tumors. Based on this classification system, we identified patients with unfavorable outcome and provided precise treatment recommendation.

## Methods

### Clinical and molecular data

The pancan.merged.V0.2.8.MC3 maf file generated by the PanCancer Atlas consortium available at the University of California Santa Cruz (UCSC) Xena browser (https://gdc.xenahubs.net) was used in this study. Meanwhile, GISTIC2 thresholded CNV data, DNA methylation (Methylation450K) data, RNA-Seq data (transcripts per kilobase million [TPM] values) transformed into log_2_(TPM + 0.001), and clinical information were also obtained from the website (https://gdc.xenahubs.net). According to the mutation information, we summarized *TP53mut* patients available in both CNV and methylation cohorts for further study. The expression and clinical data of ICGC were downloaded from ICGC (https://dcc.icgc.org/). The mutation data of ICGC was obtained from UCSC. Expression profile data (TPM values) and somatic mutation data of human CCLs were downloaded from the Broad Institute Cancer Cell Line Encyclopedia (CCLE) project (https://portals.broadinstitute.org/ccle/) (45). Drug sensitivity data of CCLs were achieved from the Cancer Therapeutics Response Portal (CTRP v.2.0, https://depmap.org/portal/download/) and the Genomics of Drug Sensitivity in Cancer (GDSC, https://www.cancerrxgene.org/). Both cohorts provided the area under the dose-response curve (AUC) values as a measure of drug sensitivity. We applied KNN imputation to impute the missing AUC values and removed compounds with more than 20% of missing data in each data set. For ICB treatment cohort, we selected the IMvigor210 cohort for further validation. We collected *TP53mut* patients and downloaded their RNA-Seq (count values) data with clinical information, and we transformed it into TPM values from the IMvigor210CoreBiologies R package. The log_2_(TPM + 0.001) was calculated with expression data for further comparison. Based on the study of cancer immunity landscape, we summarized 78 immune checkpoints molecules (stimulatory or inhibitory) for our study (Supplemental Table 4) (46).

### Single-platform clustering analyses

#### Somatic mutation clustering.

For mutation profile, we utilized the SomaticSignatures package for R to implement an algorithm that performed the nonnegative matrix factorization (NMF) to decompose the original *TP53mut* matrix to the minimal set of mutation signatures. This algorithm estimated the contribution of each signature across the samples. Rank = 5 was selected as the promising threshold where the magnitude of the cophenetic correlation coefficient began to fall, generating 5 mutational signatures (Supplemental Figure 3A). Strong correlations between the 5 signatures and COSMIC signatures (*R* = 0.99 between signature 1 and COSMIC signature 13; *R* = 0.94 between Signature5 and COSMIC signature 6), which were curated on somatic mutations from different cancers, suggesting that the 5 signatures closely related to tumor malignancies (Supplemental Figure 3, B and C). Unsupervised hierarchical clustering using Euclidean distance and Ward.D2’s linkage based on the 5 signatures to identify samples that shared similar mutational spectra classified the *TP53mut* samples into 6 clusters chosen by eclust R package with various mutational patterns (Supplemental Figure 3, D and E).

#### CNV clustering.

For somatic copy-number alterations profile, 84 focal amplification or deletion loci have been well established in regulating tumorigenesis from literature (Supplemental Table 1) (47). According to GISTIC2.0 results, we transformed these fragments’ amplification values of 1 and 2 into 1 and deletion values of –1 and –2 into –1. According to these 3 categorical variables (–1, 0, and 1), we performed unsupervised hierarchical clustering using Euclidean distance and Ward.D2’s linkage and classified *TP53mut* samples into 13 CNV groups (Supplemental Figure 4, A and B).

#### DNA methylation clustering.

For DNA methylation profile, we first selected a total of 6,085 driver CpG sites with standards as follows: (a) the mean β value < 0.2 in normal tissues and blood cells and β value > 0.3 in no more than 5 samples; (b) CpG sites methylated at a β value of > 0.3 in more than 10% of tumors; and (c) elimination of the probes on the sex chromosome and existed in single nucleotide polymorphism (SNP) alterations. To maximize the variations of each subtype clustered by CpG sites, we finally filtered the most 1,000 viable sites for unsupervised clustering using Euclidean distance and Ward.D2’s linkage. We chose 6 clusters for the subsequent clustering by eclust (Supplemental Figure 5, A and B).

### Integrated platform analysis

Clusters identified from individual platforms (Mutation, SCNA, and DNA methylation) were coded into binary variables for each platform-specific cluster, with samples belonging to the particular platform/cluster having a value of 1 and other samples having a value of 0. The matrix the values of 1 and 0 that occur multiple times was then used as the input data matrix in the ConsensusClusterPlus R package to identify integrated relationships for the 2,773 patient samples, with Euclidean distance and average linkage. We finally identified 5 clusters for the optimal clustering according to the relative change in the area under the empirical cumulative distribution function (CDF) curve (Supplemental Figure 6, A and B).

### Identification of genomic determinants of the 5 COCA subtypes

When exploring driver genes on mutation level, MafCompare was utilized to detect differentially mutated genes by performing Fisher’s exact test on all genes in the MAF files between each cohort. We finally selected the high or low SMGs as the potential driver genes (*P* < 0.001).

On somatic copy-number alterations level, we first selected the significant amplification or deletion peaks containing known oncogenes or tumor-suppressor genes. For amplification fragments, we transformed the value of –1 into 0, generating a binary variable (1 and 0). For deletion fragments, we transformed the value of 1 into 0, also generating a binary variable (1 and 0). We then separately made comparisons on these 2 binary variables between each group. Amplification or deletion fragments significantly and consistently enriched in 1 COCA group were considered as the possible driver fragments (*P* < 0.001). Finally, only oncogenes or tumor suppressor genes on these candidate fragments with consistent gene expression alterations were screened as the SCNA drivers (*P* < 0.05).

On a methylation level, before comparison between each cluster, we first dichotomized the data using a β value of ≥ 0.3 to define positive DNA methylation and < 0.3 to specify lack of methylation. We then set up a 2-step standard to choose potential driver genes on the 1,000 most viable sites: (a) genes with methylation after dichotomizing existing statistics differences during multiple comparisons between every 2 clusters by χ^2^ tests were selected (*P* < 0.001), and (b) we selected the methylated genes that negatively regulated their mRNA expression (*P* < 0.05).

### WGCNA

The WGCNA R package was utilized to establish coexpression networks of genes (48). We obtained expression profile data and a phenotype data matrix containing 2,628 samples; 19,765 genes; and 5 phenotypes. We calculated the variance of each gene in each sample and selected the criteria of genes with SD greater than 2.0 for further study. According to the correlation coefficient between gene i and j defined as Sij: Sij = |cor(i,j)|, we chose β = 6 as the soft-thresholding power to transform the coexpression similarity matrix into the adjacency matrix (Supplemental Figure 8A). Subsequently, a topological matrix was created using the topological overlap measure (TOM). We then used the average-linkage hierarchical clustering method to cluster genes (Supplemental Figure 8B). After determining the gene modules by the dynamic hybrid cut method, we calculated the module eigengenes (ME) of each module and then performed cluster analysis on the modules to merged the closer modules into a new module. At last, 13 distinct coexpression modules were identified that contained 43–1,935 genes per module (Supplemental Figure 8C). To determine the significance of each module, we calculated gene significance (GS) to measure the correlation between genes and sample traits. Module membership (MM) was defined as the average GS of genes within 1 module. We finally screened out the hub genes in 5 clinically significant modules.

### Evaluation of tumor microenvironment infiltration patterns

Scores of the 7 steps of cancer-immunity cycle and various types of immune cell infiltration were calculated by tracking tumor immunophenotype (TIP) (http://biocc.hrbmu.edu.cn/TIP/index.jsp) (49). Also, quanTIseq algorithm was used to calculate proportions of immune cells. Besides, the calculated results by xCell algorithm were obtained from xCell website (http://xcell.ucsf.edu) (50).

### Machine learning predictions

TCGA RNA-Seq cohort was used to develop classification model for the prediction of patients or cells in COCA3 or COCA1.2.4.5 subtype from the ICGC and IMvigor210 cohort or CCLE cohort and GL261 cells by machine learning. Patients were first divided into training and validation sets with a proportion of 80%:20%. The training set was then used to select characteristic variables by lasso regression for training classification model.

#### Prediction of COCA subtypes of TP53mut patients.

We first used lasso regression with the optimal α value (α = 0.0015) on TCGA mRNA-Seq cohort selecting 489 characteristic genes through an internal layer of 10,000 runs of a 5-fold cross-validation process. Then, artificial neural network, SVM, logistic regression (LR), KNN, LDA, and NB algorithms were applied to predict the different subtypes.

For artificial neural network, it was constructed based on keras with random starting parameters. The input of the first fully connected layer is the expression of 489 characteristic genes, and the output is 500 neurons. The input of layer 2 is 500 and the output is 1,500 neurons. The input of layer 3 is 1,500 and the output is 1,700 neurons. The input of layer 4 is 1,700 neurons, and the output is 2 categories. The important parameters for the model settings are as follows: batchsize = 200, epochs = 50, optimizer = Adam, learning rate = 0.001.

#### Prediction of COCA3 and COCA1.2.4.5 subtypes of CCLEs and GL261 cells from TCGA.

For CCLE cohort, considering the difference between single tumor cell and tumor tissue, we first performed debatch effects on the expression profiles of TCGA and CCLE or sequenced GL261 cells. Then, we selected 517 characteristic genes with the optimal α = 0.00015 in CCLE and 649 characteristic genes with the optimal α = 0.00012. We used the representative genes’ expression from TCGA to predict the 2 subtypes in CCLEs and GL261 cells. Finally, the neural network was selected as the optimal prediction method for the highest prediction accuracy among the 6 methods (100% in training cohort and 100% in validation cohort).

### Cell cultures and materials

LUSC cell lines NCI-H520 (iCell-h238) and NCI-H1703 (iCell-h262) and breast invasive carcinoma (BRCA) cell lines MDA-MB-231 (iCell-h133), HCC1937 (iCell-h327), and MDA-MB-468 (iCell-h138) were bought from iCell Bioscience Inc. GL261 was obtained from American Type Culture Collection. Human mononuclear macrophage line (THP-1) was purchased from the Shanghai Cell Bank of the Chinese Academy of Sciences (Shanghai, China). THP-1, HCC1937, and the LUSC tumor cells were maintained in RPMI 1640 medium (RPMI; 61870036, Thermo Fisher Scientific) containing 10% FBS (FBS, 16140071, Thermo Fisher Scientific) and 1% penicillin/streptomycin (10378016, Thermo Fisher Scientific). MDA-MB-231 and MDA-MB-468 cells were cultured in L-15 medium (11415064, Thermo Fisher Scientific), and GL261 cells were cultured in DMEM (10566024, Thermo Fisher Scientific) combined with 10% FBS and 1% penicillin/streptomycin. Olaparib (HY-10162) and doxorubicin (HY-15142A) were bought from MedChemExpress (MCE). The anti–mouse PD-1 (BE0146) was bought from Bio X Cell.

### RNA isolation and RNA-Seq

Total RNA from GL261 cells were isolated by Trizol and sent to Novogene Company (Beijing, China) for RNA-Seq. We finally transformed the count data into the log_2_(TPM + 0.001) for COCA subtype prediction.

### MTS assay

Cell proliferation was measured by the MTS assay. Five cancer cells were separately calculated and plated in 96-well plates at a cell density of 10,000 cells/well incubated with different concentration of olaparib and doxorubicin. Each group was made in triplicate. MTS reagent was added on the fourth day to estimate the live cells. Cell viability was determined by measuring the absorption at 490 nm.

### Coculturing system

Coculturing system was established by 6-well transwells (3450, Corning). The THP-1 cells were induced into THP-1–derived macrophages by 5 nM PMA (P1585, MilliporeSigma) in the bottom chambers. After 24 hours, different subtypes of BRCA and LUSC tumor cells were seeded into the top transwell chambers for 48 hours of coculture. Finally, THP-1–derived macrophages in the bottom chambers were collected for PCR and Flow cytometry tests.

### Quantitative PCR (qPCR)

After coculture management, total RNA from THP-1–derived macrophages was extracted by TaKaRa MiniBEST Universal RNA Extraction Kit. NanoDrop 2000 was used to qualify the RNA concentration. Extracted RNA was then reverse transcribed to cDNA in a 20 μL reaction system. The qPCR reactions were performed as follows: denaturation at 95°C for 30 seconds, 50 cycles of 95°C for 5 seconds per cycle, and 60°C for 30 seconds. Each sample was divided in triplicate. 18s was identified as internal control to calculated relative expression of other mRNA via the 2^–ΔΔCT^ method. PCR primer sequences showed as follows: CD11C (forward primer: 5′-GATGCTCAGAGATACTTCACGGC-3′, reverse primer: 5′- CCACACCATCACTTCTGCGTTC-3′); ARG1 (forward primer: 5′-TCATCTGGGTGGATGCTCACAC-3′, reverse primer: 5′- GAGAATCCTGGCACATCGGGAA-3′); iNOS (forward primer: 5′-CCCTTCCGAAGTTTCTGGCAGCAGCAGC-3′, reverse primer: 5′-GGCTGTCAGAGCCTCGTGGCTTTGG-3′); and 18s (forward primer: 5′-GCAGAATCCACGCCAGTACAAGAT-3′, reverse primer: 5′-TCTTCTTCAGTCGCTCCAGGTCTT-3′).

### Flow cytometry

The detailed protocol of flow cytometry was performed as previously described (51). FACS data were analyzed through FlowJo software (version 10.4).

### Cas9/CRISPR gene KO in GL261 cells

Design and synthesis of PTEN sgRNA were based on GeneChem (www.genechem.com.cn). KO of PTEN was conducted in GL261 cells by transfecting the sgRNA-expressing plasmid of PTEN or control scrambled sg (Sr-sg). Two days after transfection, the cells were treated with 4 μg/mL puromycin, and the residual resistant cells were then picked up for monoclonal screening. Finally, positive clones were selected by Western blot to confirm their KO.

### Western blot

WT and transfected GL261 cells were lysed by mixture of RIPA buffer and PMSF at the concentration of 10:1. Equal amounts (20 μg) of different proteins were put in 10% gel for electrophoresis and then transferred to polyvinylidene fluoride (PVDF) membranes. After being blocked with 5% milk/Tris-buffered saline plus Tween 20 (TBST), membranes were incubated with primary antibodies against PTEN (1:2,000; 22034-1-AP, Proteintech) and GAPDH (1:20,000; 10494-1-AP, Proteintech) at 4°C overnight. The second day, TBST was added to wash primary antibodies followed with HRP goat anti–rabbit IgG (1:5,000; 15015, Proteintech) as a secondary antibody incubation of 1 hour. Immunoreactive bands were visualized by Tanon 5200. GAPDH antibody was defined as internal control, and grayscale analysis was performed by ImageJ (NIH). Each group was divided in triplicate.

### IHC

The paraffin-embedded tissues was obtained from tumor-bearing mice under PBS or olaparib treatment. During a graded series of alcohol dewaxing and rehydration, antigen retrieval was conducted in citrate buffer at pH 6 for 2 minutes. Then, 3% H_2_O_2_ was used to block the endogenous peroxidase for 12 minutes at room temperature. Primary antibodies against Ki67 (ab15580, Abcam) and CD206 (24595, Cell Signaling Technology) were added at 4°C followed by overnight incubation and then incubated with secondary antibody. Stained tissues were imaged with a light microscope (Nikon Eclipse TS100, Olympus Optical Co.). The results were independently analyzed by 2 investigators.

### In vivo experiments

#### S.c. tumorigenic mouse model.

Four- to 6-week-old male BALB/c nude mice were bought from Beijing Vital River Laboratory Animal Technology. In total, 5 × 10^6^ cancer cells were separately injected s.c. into the right flanks of nude mice. The tumor-bearing mice were randomly divided into the control and the olaparib treatment groups (*n* = 4), respectively. When tumor reaches 50–110 mm^3^, i.p. administration of 10 mg/kg olaparib was initiated and carried out 3 times weekly for 2 weeks. Finally, the tumors were measured using calipers, and tumor volumes were calculated using the formula as follows: ([4/3] × 3.14159) × ([Length/2] × [Width/2]^2^). Moreover, the mice were sacrificed and the tumors were further fixed for Ki67 staining.

#### Intracranial mouse model.

Four- to 6-week-old male C57BL/6 mice were purchased from Beijing Vital River Laboratory Animal Technology. In total, 5 × 10^5^ GL261 WT or GL261 sgPTEN cells were injected into the right nigrostriatum of mice by using stereotaxic apparatus. Then, the mice were randomly grouped and divided into 4 groups: control (treated with IgG2b isotype control), olaparib group (treated with olaparib alone), anti–PD-1 group (treated with anti–PD-1 Ab alone), and combined group (treated with olaparib and anti–PD-1 Ab). Olaparib was injected i.p. (10 mg/kg) 3 times weekly for 2 weeks after tumor cells were inserted for 5 days. Anti–PD-1 Ab was administrated i.p. on days 5, 8, and 11 at a dose of 10 μg/g body weight. The mice were sacrificed at the same time for H&E and IHC staining or observed until death for survival analysis. All mice were kept in specific pathogen–free (SPF) conditions.

### Other bioinformatic analysis

Heatmaps were created by R to distinguish distinct group information. “Circos” R package was used to visualize the driver events under *TP53mut*. Survival and survminer R packages were applied to illustrate prognostic conditions. GO analyses were carried out using the ClueGO to explore the functional implications associated with 5 subtypes (52). Stimulatory and inhibitory scores were estimated by ssGSEA algorithm. “UpsetR” package was used to visualize the intersections between immune cells in different subtypes. GSEA was conducted to find differences in phenotypes between different subtypes (53). Potential ICB response was predicted with TIDE algorithm (54). Samples drug sensitivity prediction was performed by R package “pRRophetic” by ridge regression. The 5 subtypes’ different features were depicted by sankey diagram on Echarts online (https://echarts.apache.org/) and pie chart on Highcharts (https://www.highcharts.com.cn/).

### Data sharing statement

The gene expression, mutation, CNV, and methylation profiles and associated clinical information of patients in this study can be obtained on UCSC website (https://xenabrowser.net/datapages/). The transcriptomic data of the GL261 cells are available from GEO (GSE214398).

### Statistics

R 4.0.2 and python 3.9 were mainly utilized for statistical analysis. Either parametric test (2-tailed Student’s *t* test or 1-way ordinary ANOVA) or nonparametric test (Wilcoxon rank-sum test or 1-way Welch’s ANOVA), when data were abnormally distributed, was used to assess a continuous variable in 2 or more groups. Pearson’s χ^2^ test was used for independence tests for categorical data. Kaplan-Meier survival analysis was performed to evaluate the prognostic value. Univariate and multivariate Cox regression analyses were used to verify COCA3 independent prognostic factors. A *P* value less than 0.05 was considered significant. Data are shown as mean ± SD.

### Study approval

The experimental protocol was approved by the Ethics Committee of The First Hospital of China Medical University. Animal experiments were conducted in accordance with the China Medical University Animal Care and Use Committee guidelines and were approved by the IRB of The First Hospital of China Medical University.

## Author contributions

Conception and design were contributed by XC, TL, JW, PC, WC, and AW; data download and curation were contributed by CZ, GG, XR, and CL; methodology was contributed by PC, CZ, and QG; and manuscript writing and revision were contributed by XC, TL, JW, WC, and AW. XC, TL, and JW contributed equally to this article. All authors read and approved the final manuscript.

## Supplementary Material

Supplemental data

## Version 1 10/18/2022

In-Press Preview

## Version 2 12/08/2022

Electronic publication
