# Supplementary material for: Molecular profiling identifies distinct subtypes across TP53 mutant tumors
Source: JCI Insight. 2022 Dec 8;7(23):e156485. doi: 10.1172/jci.insight.156485 (PMC9746906; doi:10.1172/jci.insight.156485)
Supplement: Supplemental data [file jciinsight-7-156485-s140.pdf]

Supplementary Tables

Supplementary Table 1. 84 focal amplification or deletion loci in regulating tumorigenesis.

Amplification	Deletion
1p34.2	1p36.23
1p22.3	1p36.11
1q21.3	1p13.2
1q32.1	2q22.1
1q44	2q37.3
2q31.2	3p26.3
3q26.2	3p14.2
4p16.3	4p16.3
4q12	4q22.1
5p15.33	4q34.3
5q35.3	5q11.2
6p22.3	5q21.3
7p11.2	6p25.3
7q36.3	6q21
8p11.23	6q26
8p11.21	7p22.3
8q24.21	7q31.1
9p13.3	7q36.1
10p15.1	8p23.2
10q22.2	8p21.2
11q13.3	9p24.1
12p13.33	9p13.1
12q14.1	9q21.3
12q15	9q34.3
13q34	10p15.3
14q13.2	10q23.31
15q26.3	10q26.3
17q12	11p15.5
17q25.1	11q22.3
19p13.12	11q25
19q12	12p13.1
20q11.21	12q23.1
20q13.33	12q24.33
Xq28	13q11
	13q14.2
	14q24.1
	15q11.2

15q15.1
16p13.3
16q23.1
17p12
17q11.2
18q23
19p13.2
19q13.32
19q13.43
20p12.1
21q11.2
22q13.32
Xp21.2

Supplementary Table 2. The number of available omics data in different tumor types.

	CNV	SNP	Methylation
BLCA	197	199	199
BRCA	264	264	208
COAD	173	173	165
ESCA	158	158	158
GBM	92	93	36
HNSC	353	357	353
LGG	248	248	248
LIHC	108	109	108
LUAD	257	259	233
LUSC	398	401	296
PAAD	108	108	106
PRAD	57	58	58
READ	76	77	74
SARC	83	85	85
SKCM	61	70	70
STAD	212	214	194
UCEC	178	178	159
UCS	51	52	52

Supplementary Table 3. The univariant and multivariant Cox analysis of COCA3 subtype in different tumor types.

LGG

Viable	Univariate Regression (OS)		Multivariate Regression (OS)		Univariate Regression (DSS)		Multivariate Regression (DSS)	
	HR	P value	HR	P value	HR	P value	HR	P value
Cluster (COCA3vsCOCA1.2.4.5)	3.711	0.002	2.721	0.027	4.607	0.00038	3.439	0.00709

Age (Old>=60 vs Young<60)	0.850	0.823			0.958	0.952		
Gender (Female vs Male)	0.951	0.851			0.961	0.889		
Histological grade (G3vsG2)	2.023	0.010	1.740	0.055	2.065	0.0137	1.703	0.08633

LUSC

Viable	Univariate Regression (OS)		Multivariate Regression (OS)	
	HR	P value	HR	P value
Cluster (COCA3vsCOCA1.2.4.5)	1.702	0.0069	1.656	0.0105
Age (Old>=60 vs Young<60)	1.557	0.11		
Gender (Female vs Male)	1.557	0.11		
Pathologic tumor stage (III-IVvsI-II)	1.712	0.0144	1.638	0.0249

STAD

Viable	Univariate Regression (OS)		Multivariate Regression (OS)		Univariate Regression (DSS)		Multivariate Regression (DSS)	
	HR	P value	HR	P value	HR	P value	HR	P value
Cluster (COCA3vsCOCA1.2.4.5)	3.265	0.0233	3.081	0.03208	5.922	0.000891	5.939	0.00109
Age (Old>=60 vs Young<60)	1.460	0.191			1.109	0.758		
Gender (Female vs Male)	1.017	0.168			0.995	0.746		
Histological grade (G3vsG1-G2)	1.360	0.227			1.248	0.479		
Pathologic tumor stage (III-IVvsI-II)	2.268	0.0019	2.241	0.00219	2.268	0.00186	2.912	0.00184

Supplementary Table 4. The list of immune checkpoint associated genes.

HGNC Symbol	Gene Family	Super Category	Immune Checkpoint
ADORA2A	Receptor	Receptor	Inhibitory
ARG1	Enzyme	Other	Inhibitory
BTLA	Immunoglobulin	Receptor	Inhibitory
CD274	B7/CD28	Co-inhibitor	Inhibitory
CD276	B7/CD28	Co-inhibitor	Inhibitory
CTLA4	Receptor	Receptor	Inhibitory
EDNRB	Receptor	Receptor	Inhibitory
HAVCR2	Immunoglobulin	Receptor	Inhibitory
IDO1	Enzyme	Other	Inhibitory
IL10	Cytokine	Ligand	Inhibitory
IL13	Cytokine	Ligand	Inhibitory
IL4	Cytokine	Ligand	Inhibitory
KIR2DL1	KIR	Receptor	Inhibitory
KIR2DL2	KIR	Receptor	Inhibitory
KIR2DL3	KIR	Receptor	Inhibitory

LAG3	Immunoglobulin	Receptor	Inhibitory
PDCD1	B7/CD28	Receptor	Inhibitory
SLAMF7	SLAM	Co-inhibitor	Inhibitory
TGFB1	Cytokine	Ligand	Inhibitory
TIGIT	PVR	Receptor	Inhibitory
VEGFA	Growth factor	Ligand	Inhibitory
VEGFB	Growth factor	Ligand	Inhibitory
C10orf54	Immunoglobulin	Co-inhibitor	Inhibitory
VTCN1	B7/CD28	Co-inhibitor	Inhibitory
GZMA	Granzyme	Other	Stimulatory
BTN3A1	Butyrophilins	Co-inhibitor	Stimulatory
BTN3A2	Butyrophilins	Co-inhibitor	Stimulatory
CCL5	Chemokine	Ligand	Stimulatory
CD27	TNFR	Receptor	Stimulatory
CD28	B7/CD28	Co-stimulator	Stimulatory
CD40	TNFR	Receptor	Stimulatory
CD40LG	TNF	Ligand	Stimulatory
CD70	TNF	Ligand	Stimulatory
CD80	B7/CD28	Co-stimulator	Stimulatory
CX3CL1	CXC chemokine	Ligand	Stimulatory
CXCL10	CXC chemokine	Ligand	Stimulatory
CXCL9	CXC chemokine	Ligand	Stimulatory
ENTPD1	Ectonucleotidase	Other	Stimulatory
HMGB1	HMG-box	Other	Stimulatory
ICAM1		Cell adhesion	Stimulatory
ICOS	B7/CD28	Receptor	Stimulatory
ICOSLG	Ligand	Co-stimulator	Stimulatory
IFNA1	Cytokine	Ligand	Stimulatory
IFNA2	Cytokine	Ligand	Stimulatory
IFNG	Cytokine	Ligand	Stimulatory
IL1A	Cytokine	Ligand	Stimulatory
IL1B	Cytokine	Ligand	Stimulatory
IL2	Cytokine	Ligand	Stimulatory
IL2RA	Type I cytokine receptor	Receptor	Stimulatory
ITGB2	Integrin	Cell adhesion	Stimulatory
PRF1	Pore	Other	Stimulatory
SELP	Selectin	Cell adhesion	Stimulatory
TLR4	Receptor	Receptor	Stimulatory
TNF	Cytokine	Ligand	Stimulatory
TNFRSF14	TNFR	Receptor	Stimulatory
TNFRSF18	TNFR	Receptor	Stimulatory
TNFRSF4	TNFR	Receptor	Stimulatory
TNFRSF9	TNFR	Receptor	Stimulatory

TNFSF4	TNF	Ligand	Stimulatory
TNFSF9	TNF	Ligand	Stimulatory
IL12A	Cytokine	Ligand	Stimulatory

Supplementary Table 5. The list of biomarkers for response to immune checkpoint therapy that were compared in this study.

IFNG.Sig	CD8.Sig	PDL1.Sig	IMPRES.Sig
IFNG	CD8A	PDL1	BTLA
STAT1	CD8B	PDCD1LG2	CD200
IDO1	CD3D	PDCD1	CD200R1
CXCL10	CD3E		CD27
CXCL9	CD3G		CD276
HLA-DRA			CD28
			CD40
			CD80
			CD86
			CEACAM1
			CTLA4
			IDO1
			IL2RB
			LAG3
			PVR
			PVRL2
			TIGIT
			TNFRSF18
			TNFRSF4
			TNFRSF9
			PDL1
			HAVCR2
			PDCD1
			PDCD1LG2
			TNFRSF14
			TNFSF4
			TNFSF9
			C10orf54

Supplementary Table 6. The target pathway associated genes of the 9 small molecule perturbagens.

PI3K-AKT-mTOR signaling pathway	DNA replication	Signaling by Receptor Tyrosine Kinases	DNA repair pathways	HISTONE_METHYLTRANSFERASE_COMPLEX	PPARγ signal pathway
TFEB	CDC45	AKT3	POLD1	AEBP2	PEPC

AKT1	CDC6	ABI1	PCNA	ASH2L	AQP7
HRAS	CDC7	SH2B3	RPA2	BOD1	CYP8B1
PTEN	CDK2	CDH5	RPA3	BOD1L1	CYP7A1
GSK3B	CDT1	RANBP9	POLD2	C17orf49	CYP27
NOS3	DBF4	PTPRU	POLD4	CBX5	NR1H3
CDKN1B	GMNN	DNAL4	MSH2	CHD8	ME1
NRAS	MCM10	ABI2	LIG1	CXXC1	SCD
PIK3CG	MCM2	WASF2	POLD3	DPY30	FADS2
ULK1	MCM3	RNF41	RPA1	DYDC1	UCP1
PIK3R3	MCM4	ADAM10	MSH6	DYDC2	CAP
MTOR	MCM5	CDK5	MSH3	E2F6	MMP1
KRAS	MCM6	TRIB1	ERCC1	EED	ADIPOQ
PIK3CA	MCM7	SPRY1	ERCC4	EZH1	PGAR
PIK3CB	ORC1	SPRY2	GTF2H1	EZH2	AP2
PIK3R1	ORC2	STAM2	GTF2H2	HCFC1	PLIN1
PIK3R2	ORC3	STUB1	GTF2H3	HCFC2	KLK15
FOXO3	ORC4	TCIRG1	POLE	HDAC2	SCP2
ATG13	ORC5	LAMC3	XPA	HDAC9	CPT2
TSC2	ORC6	YAP1	ERCC8	INO80C	CYP4A1
FOXO4	PCNA	VAV3	ERCC5	JARID2	LCAD
FOXO1	POLA1	BAIAP2	ERCC6	KANSL1	CPT1
EIF4EBP1	POLA2	SH2B2	RAD23B	KAT8	MCAD
GRB10	POLD1	MXD4	RFC1	KDM6A	ACBP
PDK1	POLD2	CDC37	UBC	KDM6B	FABP1
RHEB	POLD3	ADCYAP1	ARPC3	KMT2A	FABP3
RICTOR	POLD4	ADCYAP1R1	ATRIP	KMT2B	LPL
RPTOR	POLE	THEM4	BARD1	KMT2C	ACS
RB1CC1	POLE2	AP2M1	CHEK2	KMT2D	FATP1
BAD	PRIM1	AP2S1	DCLRE1A	LAS1L	FATP4
	PRIM2	CLTA	GADD45A	MAX	FARCD36
	RFC1	CLTC	GADD45B	MCRS1	OLR1
	RFC2	CMA1	PSMA4	MEN1	GK
	RFC3	ATP6V1G3	PSMA5	MGA	PPARG
	RFC4	COL1A1	PSMA6	NCOA6	
	RFC5	COL1A2	PSMA7	PAGR1	
	RPA1	SPINT2	PSMB1	PAXIP1	
	RPA2	NRG3	PSMB10	PELP1	
	RPA3	GRAP	PSMB2	PHF20	
	RPA4	AHCYL1	PSMB3	PRDM4	
	UBA52	NCKAP1	PSMB4	PRMT5	
	UBC	WASF3	PSMB5	PRPF31	
	POLD1	FRS3	PSMB6	RBBP4	

PCNA	FRS2	PSMB7	RBBP5
RPA2	ADAP1	PSMB8	RBBP7
RPA3	WWP1	PSMB9	RNF2
POLD2	CHD4	PSMC1	RUVBL1
POLD4	CHEK1	PSMC2	RUVBL2
MSH2	COL9A1	PSMC3	SENP3
LIG1	COL9A2	PSMC4	SETD1A
POLD3	COL9A3	PSMC5	SETD1B
RPA1	COL11A1	PSMC6	SUZ12
MSH6	COL11A2	PSMD1	TAF1
MSH3	COL6A6	PSMD2	TAF4
ERCC1	ADORA2A	PSMD3	TAF6
ERCC4	CREB1	PSMD4	TAF7
GTF2H1	ATF2	PSMD5	TAF9
GTF2H2	CRK	PSMD7	TEX10
GTF2H3	CRKL	RAD1	UTY
POLE	AAMP	RAD17	WDR5
XPA	COL2A1	RAD9A	WDR5B
ERCC8	COL3A1	POLR1D	WDR82
ERCC5	COL4A1	TP53	
ERCC6	COL4A2	TREX1	
RAD23B	COL4A3	TREX2	
RFC1	COL4A4	APEX2	
RFC2	COL4A5	MUTYH	
RFC3	COL5A1	CENPX	
RFC4	COL5A2	USP1	
RFC5	COL6A1	POLI	
XPC	COL6A2	RAD51C	
ERCC2	COL6A3	REV3L	
ERCC3	AP2A1	HMGB1	
POLE2	AP2A2	UNG	
POLE4	SPRED1	H2AX	
MLH1	AP2B1	MPG	
PMS2	DIAPH1	RAP1A	
LIG3	DLG4	FAAP100	
LIG4	DNM1	TDG	
DDB1	DNM2	TERF2	
DDB2	DOCK1	PALB2	
GTF2H4	DOCK3	FANCM	
GTF2H5	HBEGF	FANCB	
POLK	DUSP3	CENPS	
POLE3	MAPK14	NEIL3	
CUL4B	FGFBP3	WDR48	

CUL4A	CSK	NEIL2
RBX1	CSN2	BRIP1
EXO1	NRG4	FAAP24
PARP1	CTNNA1	FANCL
ATM	CTNNB1	FANCI
BRCA1	CTNND1	REV1
BRCA2	KLB	FAN1
XRCC6	CYBA	SLX4IP
MRE11	CYBB	USP7
NBN	ATP6V0E2	HMGN1
POLH	EP300	UVSSA
PRKDC	EPS15	SLX4
RAD50	ERBB2	APEX1
RAD51	ERBB3	ATR
XRCC1	ERBB4	CHEK1
XRCC4	EREG	FANCA
XRCC5	AKT1	FANCC
RAD23A	AKT2	FANCD2
CDK7	ESR1	FANCE
CETN2	F3	FANCF
MNAT1	PTK2B	FANCG
CCNH	FER	FEN1
POLR1A	DUSP4	MBD4
POLR2A	DUSP6	MGMT
POLR2B	DUSP7	NTHL1
POLR2C	EGF	OGG1
PARP2	EGFR	PNKP
CHD1L	EGR1	POLB
APEX1	EGR2	SMUG1
ATR	EGR3	XAB2
CHEK1	EGR4	RAD54B
FANCA	ELK1	RAD52
FANCC	SPRED2	WRN
FANCD2	MUC20	DCLRE1C
FANCE	FGFR3	NHEJ1
FANCF	FGFR2	POLM
FANCG	FGFR4	GADD45G
FEN1	VEGFD	HUS1
MBD4	NCBP2	IGF1
MGMT	TAB2	MAPK10
NTHL1	GGA3	MAPK11
OGG1	CYFIP1	MAPK12
PNKP	FLT1	MAPK13

POLB	FLT3	MAPK14
SMUG1	FLT3LG	MAPK8
XAB2	ARC	MAPK9
RAD54B	FES	MDM2
RAD52	FGF1	PSMD6
WRN	FGF2	POLA1
DCLRE1C	FGF3	POLG
NHEJ1	FGF4	POLQ
POLM	FGF5	POLR1B
POLL	FGF6	POLR1C
RPS27A	FGF7	POLR3D
UBA52	FGF8	POLR3F
UBB	FGF9	POLR3K
UBC	FGF10	TENT4A
ARPC3	FGFR1	PSMA1
ATRIP	ATP6V1C2	PSMA2
BARD1	MTOR	PSMA3
CHEK2	RICTOR	RFC2
DCLRE1A	FYN	RFC3
GADD45A	GAB1	RFC4
GADD45B	EPGN	RFC5
GADD45G	GABRA1	XPC
HUS1	COL24A1	ERCC2
IGF1	GABRB1	ERCC3
MAPK10	COL6A5	POLE2
MAPK11	GABRB2	POLE4
MAPK12	GABRB3	MLH1
MAPK13	FLT4	PMS2
MAPK14	FN1	LIG3
MAPK8	NCSTN	LIG4
MAPK9	FOS	DDB1
MDM2	FOSB	DDB2
PSMD6	RBFOX2	GTF2H4
POLA1	ATP6V0A2	GTF2H5
POLG	FLRT3	POLK
POLQ	FLRT2	POLE3
POLR1B	FLRT1	CUL4B
POLR1C	ALK	CUL4A

POLR3D	ATP6V0D2	RBX1
POLR3F	FGF22	EXO1
POLR3K	LAMA1	PARP1
TENT4A	GRB2	ATM
PSMA1	GRB7	BRCA1
PSMA2	GRB10	BRCA2
PSMA3	RAPGEF1	XRCC6
PSMA4	GRIN2B	MRE11
PSMA5	CD274	NBN
PSMA6	GTF2F1	POLH
PSMA7	GTF2F2	PRKDC
PSMB1	EPN1	RAD50
PSMB10	SH3KBP1	RAD51
PSMB2	GABRG2	XRCC1
PSMB3	GABRG3	XRCC4
PSMB4	SHC2	XRCC5
PSMB5	GALNT3	RAD23A
PSMB6	NELFB	CDK7
PSMB7	SIN3A	CETN2
PSMB8	LRIG1	MNAT1
PSMB9	DNM3	CCNH
PSMC1	FGF20	POLR1A
PSMC2	PTPN18	POLR2A
PSMC3	GFAP	POLR2B
PSMC4	CYFIP2	POLR2C
PSMC5	HRAS	PARP2
PSMC6	HSPB1	CHD1L
PSMD1	HSP90AA1	POLL
PSMD2	ID1	RPS27A
PSMD3	ID2	UBA52
PSMD4	ID3	UBB
PSMD5	ID4	
PSMD7	IGF1	
RAD1	APOE	
RAD17	IGF1R	
RAD9A	IGF2	
POLR1D	IL2RG	
TP53	HDAC1	
TREX1	HDAC2	
TREX2	NCKAP1L	
APEX2	HGF	
MUTYH	HGFAC	
CENPX	NRG1	

USP1	PIK3R4
POLI	HIF1A
RAD51C	HNRNPA1
REV3L	HNRNPF
HMGB1	HNRNPH1
UNG	HPN
H2AX	JAK3
MPG	JUNB
RAP1A	JUND
FAAP100	JUP
TDG	ANOS1
TERF2	AREG
PALB2	KDR
FANCM	KIT
FANCB	ARF6
CENPS	KRAS
NEIL3	RHOA
WDR48	LAMA2
NEIL2	INS
BRIP1	INSR
FAAP24	IRS1
FANCL	ITGA2
FANCI	ITGA3
REV1	ITGAV
FAN1	ITGB1
SLX4IP	ITGB3
USP7	ITPR1
HMGN1	ITPR2
UVSSA	ITPR3
SLX4	JAK2
	MATK
	MDK
	MEF2A
	MEF2C
	MEF2D
	MET
	KITLG
	ASCL1
	MMP9
	MST1
	MST1R
	MYC
	LAMA3

LAMA4
LAMA5
LAMB1
LAMB2
LAMB3
LAMC1
LAMC2
STMN1
LCK
LYL1
LYN
NRAS
NTF3
NTF4
NTRK1
NTRK2
NTRK3
FURIN
PCSK6
COL5A3
PAK1
ATP6V0A4
PAK2
MYCN
ATF1
NAB1
NAB2
HNRNPM
NCBP1
NCF2
NCF4
NCK1
NEDD4
NGF
NOS3
PDPK1
WWOX
PGF
ATP6V1A
PGR
ATP6V1B1
ATP6V1B2
ATP6V0C

ATP6V1C1
PIK3C3
ATP6V1E1
PIK3CA
PAK3
MEMO1
APH1A
VRK3
PCSK5
ATP6V1D
PDE3B
PDGFA
PDGFB
PDGFRA
PDGFRB
ATP6V1H
RAB4B
TLR9
POLR2A
POLR2B
POLR2C
POLR2D
POLR2E
POLR2F
POLR2G
POLR2H
POLR2I
POLR2J
PIK3CB
PIK3R1
PIK3R2
PLAT
ATP6V0B
PLCG1
SHC3
ATP6V1G2
PLG
ATP6V0A1
ATP6AP1
FGFRL1
PRKCA
PRKCB
AXL

PRKCD
PRKCE
PAG1
BRK1
PSENEN
GABRQ
PRKCZ
ERBIN
MAPK1
POLR2K
POLR2L
ESRP1
PPP2CA
PPP2CB
PPP2R1A
PPP2R1B
PPP2R5D
PRR5
PRKACA
PRKACB
PRKACG
KIDINS220
PTK6
RANBP10
PTN
PTPN1
PTPN2
PTPN3
TRIB3
PTPN6
PTPN11
PTPN12
PTPRJ
MAPK3
MAPK7
MAPK11
MAPK13
PDGFC
MAP2K1
MAP2K2
MAP2K5
PSEN1
PSEN2

PTBP1
PTK2
RAP1A
RAP1B
RASA1
REST
ACTB
RIT2
RIT1
ROCK1
RPS6KA1
RPS6KA2
RPS6KA3
RPS27A
PTPRK
PTPRO
PTPRS
PTPRZ1
BAX
PXN
EPS15L1
RAB4A
RAC1
RALA
RALB
RALGDS
SHB
SHC1
TNS3
NCF1
SOS1
SPARC
SPINT1
SPP1
SRC
SRF
BRAF
STAT1
RRAD
BDNF
S100B
MAPK12
CXCL12

PRDM1
ELMO2
MLST8
SGK1
SH3GL1
SH3GL2
SH3GL3
STAT3
STAT5A
STAT5B
STAT6
BTC
ADAM17
TCF12
TEC
TGFA
THBS1
THBS2
THBS3
THBS4
TIA1
TIAL1
TIAM1
ACTG1
TPH1
UBA52
UBB
UBC
VAV1
VAV2
VEGFA
VEGFB
VEGFC
VGF
YES1
YWHAB
MAPKAPK3
MAPKAP1
ESRP2
CALM1
STAM
PDGFD
ADAM12

CALM2
FOSL1
CUL5
FGF23
CALM3
APH1B
ITCH
FGFBP2
NCK2
IRS4
CILP
TNS4
NRP1
HDAC3
CDK5R1
ARHGEF7
SPHK1
WASF1
CDK5R2
ATP6V0E1
ATP6V1E2
SH2D2A
USP8
ATP6V0D1
PIK3R3
COL27A1
DOCK7
MKNK1
CAV1
SOCS1
IRS2
CBL
FGF18
FGF17
FGF16
NRP2
HGS
RPS6KA5
MAPKAPK2
ATP6V1F
SOCS6
KL
GRAP2

	ROCK2
	NRG2
	ATP6V1G1
	BCAR1
	NCOR1
	ELMO1
	GAB2
	FGF19
	CDC42
	FGFBP1

Supplementary Table 7. The predicted subtypes of TP53 mutant tumor cell lines.

Cells	Cluster
COLO-783	COCA1.2.4.5
VCaP	COCA1.2.4.5
RKN	COCA1.2.4.5
SK-UT-1	COCA1.2.4.5
8-MG-BA	COCA1.2.4.5
CAL-120	COCA1.2.4.5
NCI-H596	COCA1.2.4.5
RERF-LC-KJ	COCA1.2.4.5
UACC-893	COCA1.2.4.5
MKN7	COCA1.2.4.5
DAN-G	COCA1.2.4.5
HCC-15	COCA1.2.4.5
SK-LU-1	COCA1.2.4.5
42-MG-BA	COCA1.2.4.5
HOP-62	COCA1.2.4.5
647-V	COCA1.2.4.5
HSC-3	COCA1.2.4.5
HCC-44	COCA1.2.4.5
NCI-H2291	COCA1.2.4.5
HSC-4	COCA1.2.4.5
SNU-407	COCA1.2.4.5
MeWo	COCA1.2.4.5
UM-UC-3	COCA1.2.4.5
HCC1569	COCA1.2.4.5
HCC-78	COCA1.2.4.5
MDA-MB-231	COCA1.2.4.5
FaDu	COCA1.2.4.5
EFM-19	COCA1.2.4.5
TC-71	COCA1.2.4.5

SK-MEL-3	COCA1.2.4.5
PA-TU-8902	COCA1.2.4.5
SNU-81	COCA1.2.4.5
NCI-H1568	COCA1.2.4.5
SW948	COCA1.2.4.5
NUGC-3	COCA1.2.4.5
639-V	COCA1.2.4.5
CL-40	COCA1.2.4.5
SCC-15	COCA1.2.4.5
HUP-T3	COCA1.2.4.5
HSC-2	COCA1.2.4.5
SW620	COCA1.2.4.5
KYSE-180	COCA1.2.4.5
NCI-H2087	COCA1.2.4.5
KYSE-410	COCA1.2.4.5
J82	COCA1.2.4.5
KP-2	COCA1.2.4.5
JIMT-1	COCA1.2.4.5
HCC1599	COCA1.2.4.5
NCI-H2030	COCA1.2.4.5
NCI-H2405	COCA1.2.4.5
NCI-N87	COCA1.2.4.5
TE-6	COCA1.2.4.5
T98G	COCA1.2.4.5
OE19	COCA1.2.4.5
OE21	COCA1.2.4.5
NCI-H1975	COCA1.2.4.5
KNS-62	COCA1.2.4.5
CAMA-1	COCA1.2.4.5
PA-TU-8988T	COCA1.2.4.5
EC-GI-10	COCA1.2.4.5
Capan-1	COCA1.2.4.5
QGP-1	COCA1.2.4.5
JHH-7	COCA1.2.4.5
TGBC11TKB	COCA1.2.4.5
KYSE-140	COCA1.2.4.5
CAL-148	COCA1.2.4.5
ECC12	COCA1.2.4.5
EN	COCA1.2.4.5
PE/CA-PJ15	COCA1.2.4.5
MFE-296	COCA1.2.4.5
PSN1	COCA1.2.4.5
CAL-33	COCA1.2.4.5

NCI-H1355	COCA1.2.4.5
SNU-C5	COCA1.2.4.5
TE-9	COCA1.2.4.5
HCC1937	COCA1.2.4.5
HCC202	COCA1.2.4.5
BT-474	COCA1.2.4.5
HCC-366	COCA1.2.4.5
LOU-NH91	COCA1.2.4.5
TE-15	COCA1.2.4.5
YAPC	COCA1.2.4.5
TE-5	COCA1.2.4.5
LXF-289	COCA1.2.4.5
BFTC-905	COCA1.2.4.5
NCI-H2122	COCA1.2.4.5
CFPAC-1	COCA1.2.4.5
Calu-6	COCA1.2.4.5
SUIT-2	COCA1.2.4.5
Calu-3	COCA1.2.4.5
COLO-680N	COCA1.2.4.5
EBC-1	COCA1.2.4.5
SCC-25	COCA1.2.4.5
KNS-42	COCA1.2.4.5
MKN1	COCA1.2.4.5
BxPC-3	COCA1.2.4.5
NCI-H23	COCA1.2.4.5
NCI-H747	COCA1.2.4.5
TE-4	COCA1.2.4.5
SW837	COCA1.2.4.5
ABC-1	COCA1.2.4.5
NCI-H1781	COCA1.2.4.5
HT55	COCA1.2.4.5
T84	COCA1.2.4.5
NCI-H1838	COCA1.2.4.5
SNU-449	COCA1.2.4.5
NCI-H441	COCA1.2.4.5
SNU-C1	COCA1.2.4.5
BT-20	COCA1.2.4.5
KYSE-70	COCA1.2.4.5
NCI-H2228	COCA1.2.4.5
HCC1954	COCA1.2.4.5
NCI-H520	COCA1.2.4.5
NCI-H1792	COCA1.2.4.5
AsPC-1	COCA1.2.4.5

TE-11	COCA1.2.4.5
NCI-H508	COCA1.2.4.5
HCC1143	COCA1.2.4.5
SW1463	COCA1.2.4.5
BT-483	COCA1.2.4.5
SNU-1040	COCA1.2.4.5
NCI-H1648	COCA1.2.4.5
SNU-61	COCA1.2.4.5
HCC2218	COCA1.2.4.5
HGC-27	COCA1.2.4.5
NCI-H1869	COCA1.2.4.5
NCI-H716	COCA1.2.4.5
OCUM-1	COCA1.2.4.5
SW1417	COCA1.2.4.5
OE33	COCA1.2.4.5
RCM-1	COCA1.2.4.5
HCC1419	COCA1.2.4.5
MDA-MB-361	COCA1.2.4.5
SK-MEL-30	COCA1.2.4.5
HPAF-II	COCA1.2.4.5
LK-2	COCA1.2.4.5
RT-112	COCA1.2.4.5
KYSE-450	COCA1.2.4.5
C2BBe1	COCA1.2.4.5
CL-11	COCA1.2.4.5
TE-1	COCA1.2.4.5
KYSE-150	COCA1.2.4.5
SW1116	COCA1.2.4.5
KYSE-510	COCA1.2.4.5
EFM-192A	COCA1.2.4.5
HT-29	COCA1.2.4.5
KYSE-520	COCA1.2.4.5
KM12	COCA1.2.4.5
HCT-15	COCA1.2.4.5
HCC2157	COCA1.2.4.5
NCI-H1437	COCA1.2.4.5
CCK-81	COCA1.2.4.5
LN-18	COCA1.2.4.5
CL-34	COCA1.2.4.5
HCC-56	COCA1.2.4.5
IGR-37	COCA1.2.4.5
MFE-319	COCA1.2.4.5
RL95-2	COCA1.2.4.5

NCI-H1650	COCA3
FU97	COCA3
MDA-MB-436	COCA3
KYSE-270	COCA3
SK-ES-1	COCA3
GI-1	COCA3
SCC-9	COCA3
HCC1806	COCA3
NCI-H1623	COCA3
MDA-MB-468	COCA3
JHH-6	COCA3
RERF-LC-MS	COCA3
KLE	COCA3
TE-8	COCA3
SF539	COCA3
CAL-85-1	COCA3
NCI-H2342	COCA3
NCI-H838	COCA3
BT-549	COCA3
NCI-H2009	COCA3
TCCSUP	COCA3
CAL-29	COCA3
RH-41	COCA3
NCI-H1651	COCA3
SF268	COCA3
HOS	COCA3
EPLC-272H	COCA3
NCI-H522	COCA3
NCI-H2023	COCA3
NCI-H1693	COCA3
GMS-10	COCA3
SK-MES-1	COCA3
HCC1395	COCA3
SNU-182	COCA3
NCI-H2085	COCA3
HT-1376	COCA3
SNU-423	COCA3
huH-1	COCA3
NCI-H647	COCA3
HUP-T4	COCA3
IPC-298	COCA3
HuH-7	COCA3
SNU-475	COCA3

AU565	COCA3
5637	COCA3
HDQ-P1	COCA3
NCI-H1755	COCA3
SCC-4	COCA3
NCI-H1703	COCA3
BHY	COCA3
RERF-LC-Sq1	COCA3
RD	COCA3
MDA-MB-415	COCA3
SNU-387	COCA3
CAS-1	COCA3
22Rv1	COCA3
SNB75	COCA3
EKVX	COCA3
RERF-GC-1B	COCA3
MDA-MB-157	COCA3
ESS-1	COCA3
JHH-4	COCA3
YH-13	COCA3
HARA	COCA3
HCC38	COCA3
SK-MEL-28	COCA3
MFE-280	COCA3
NCI-H1793	COCA3
PC-3	COCA3
NCI-H2170	COCA3
TE-10	COCA3
GCT	COCA3
NCI-H1435	COCA3
JHH-2	COCA3
KALS-1	COCA3
NCI-H1734	COCA3
RVH-421	COCA3
COLO-800	COCA3
RPMI-7951	COCA3
HCC70	COCA3
GCIY	COCA3
PC-14	COCA3
NCI-H1573	COCA3
A2058	COCA3
